# Spatial multi-omics-guided drug development in inflammatory bowel disease: from mucosal immune niches to precision therapy

**DOI:** 10.3389/fimmu.2026.1951659

**Published:** 2026-09-18

**Authors:** Hai Wang, Xiaolong Zhao, Jie Yang

**Affiliations:** 1 Bazhong Central Hospital, Sichuan Academy of Medical Sciences and Sichuan Provincial People’s Hospital, Bazhong, China; 2 Department of Otolaryngology Head and Neck Surgery, Sichuan Academy of Medical Sciences and Sichuan Provincial People’s Hospital, School of Medicine, University of Electronic Science and Technology of China, Chengdu, China; 3 Department of Pharmacy, Personalized Drug Research and Therapy Key Laboratory of Sichuan Province, Sichuan Academy of Medical Sciences and Sichuan Provincial People’s Hospital, School of Medicine, University of Electronic Science and Technology of China, Chengdu, China

**Keywords:** crohn’s disease, drug development, inflammatory bowel disease, mucosal immune niches, precision medicine, spatial biomarkers, spatial multi-omics, ulcerative colitis

## Abstract

Crohn’s disease and ulcerative colitis are now treated with an expanding set of biologics and small molecules, yet treatment selection remains largely empirical. Primary non-response, secondary loss of response, incomplete mucosal healing, fibrostenosis, and fistulizing disease show that conventional clinical, endoscopic, and bulk molecular markers do not capture how disease is organized within tissue. This review argues that the inflamed intestine is better viewed as a set of spatially structured mucosal immune niches rather than as uniform inflammation, with epithelial, myeloid, lymphoid, stromal, vascular, neural, and microbiome-associated cells communicating through defined cytokine and ligand–receptor circuits. In this framework, mucosal immune niches can be considered functional tissue units that link tissue-resolved mechanisms to therapeutic target discovery, pharmacodynamic assessment, biomarker qualification, and biomarker-enriched trial design. Single-cell atlases and spatial multi-omics, interpreted through computational modeling, can identify disease-driving cellular neighborhoods, ligand–receptor circuits, and compartment-specific vulnerabilities in both diseases. We discuss how these spatial features may inform target discovery, mechanism-of-action validation, patient stratification, response prediction, and trial design, using pathways such as TNF, IL-23, OSM, TL1A, integrins, JAK–STAT, and S1P as examples. At present, the realistic value of spatial data lies in generating mechanistic hypotheses, defining pharmacodynamic endpoints, and enriching trials, not in guiding routine treatment selection. Most candidate spatial biomarkers remain at the discovery or early translational stage and require standardized sampling, harmonized computation, longitudinal assessment, and multicenter validation. A staged translational path is proposed, moving from disease-specific atlases to spatial pharmacodynamic endpoints and adaptive precision trials, while also offering a comparative framework for studying chronic inflammation across mucosal organs.

## Graphical Abstract

**Figure:**
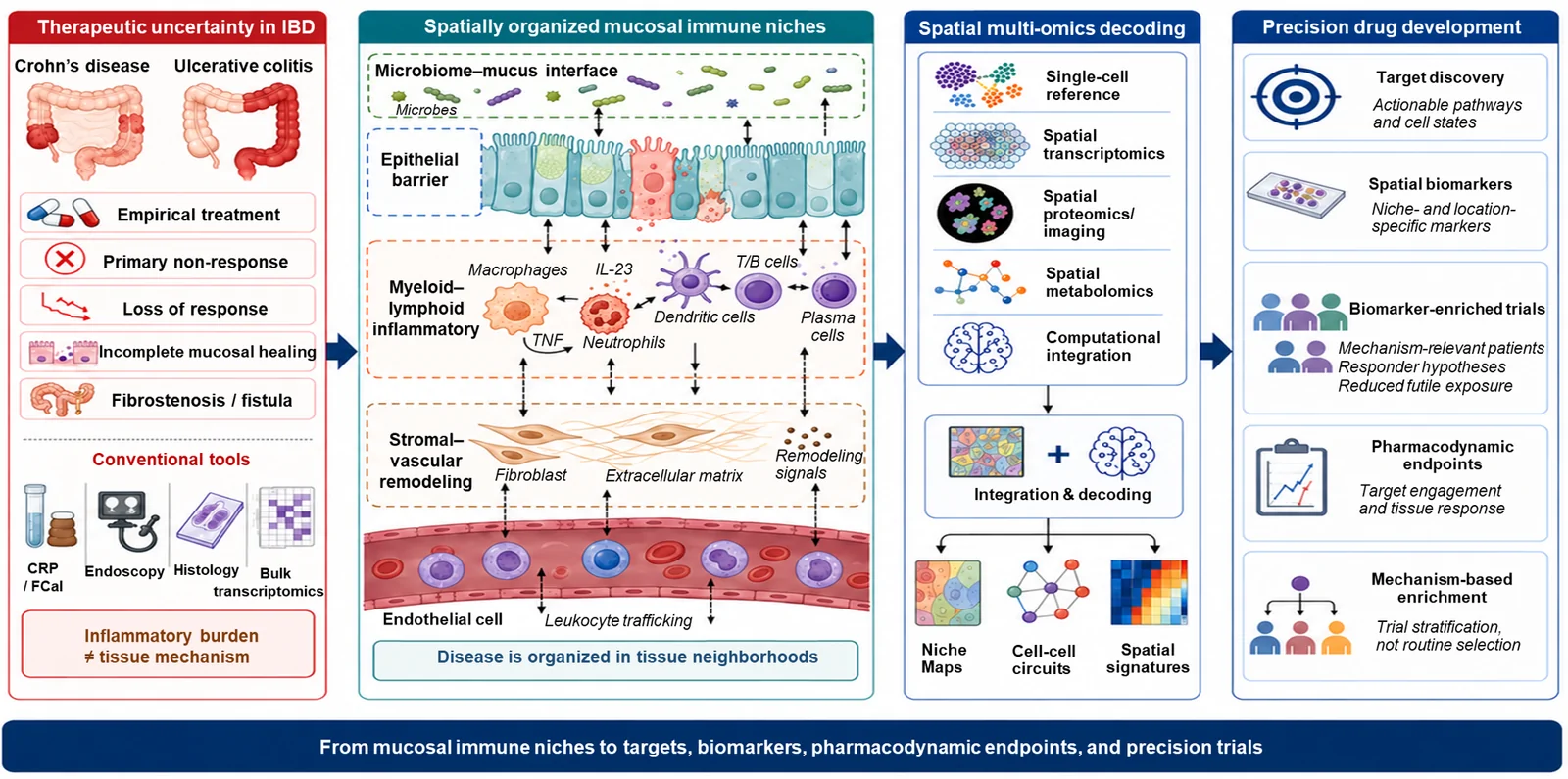
Spatial multi-omics-guided precision drug development in inflammatory bowel disease. Conventional clinical, endoscopic, histologic, and bulk molecular tools quantify inflammatory burden but do not adequately resolve the tissue organization and cellular circuits that sustain individual lesions. Integrating single-cell references with spatial transcriptomics, spatial proteomics and imaging, spatial metabolomics, and computational analysis can resolve mucosal niches and generate candidate therapeutic targets, spatial biomarkers, pharmacodynamic readouts, and signatures for trial enrichment. At present, these applications are most credible for mechanistic discovery, pharmacodynamic assessment, and trial enrichment rather than routine treatment selection.

## 1 Introduction

Crohn’s disease and ulcerative colitis affect millions of people worldwide, and their incidence continues to rise in newly industrialized regions (1, 2). Decades of work on barrier function, innate and adaptive immunity, and host–microbe interaction have produced a layered model of pathogenesis in which genetic susceptibility, environmental exposure, epithelial stress, and dysregulated mucosal immunity converge on chronic, relapsing inflammation (3–5). That understanding has supported a shift from symptom control toward treat-to-target strategies and a therapeutic landscape that now spans anti-TNF antibodies, anti-integrin agents, IL-12/23 and selective IL-23p19 inhibitors, JAK inhibitors, S1P receptor modulators, and emerging TL1A/DR3-directed therapies (6–11).

Yet more mechanisms have not produced more certainty at the bedside. Across drug classes, a substantial fraction of patients show no meaningful early benefit, and many who respond later lose that response (12–20). Anti-TNF therapy illustrates the problem most clearly, because primary non-response and immunogenic loss of response remain common despite long clinical experience, including immunogenicity captured in large prospective cohorts (21–25). The newer agents have broadened the options without resolving the central question, which is not whether a drug works in a population but which drug fits a given patient, lesion type, disease location, and disease stage. Fibrostenotic and fistulizing Crohn’s disease expose the limits of an inflammation-centered model most sharply, since these complications reflect tissue remodeling that anti-inflammatory drugs address incompletely. In practice, many patients still move through therapies in sequence, accumulating drug exposure and losing time before an effective mechanism is found, potentially delaying effective therapy or timely surgical intervention. The problem is partly one of tissue organization, not only of drug availability.

The tools used to monitor IBD measure inflammatory burden well but rarely identify mechanism. C-reactive protein and fecal calprotectin track the intensity of inflammation and correlate with endoscopic activity, yet they do not indicate which cellular circuit sustains a given lesion (26). Endoscopy and histology describe morphology and have anchored modern endpoints such as mucosal and histologic healing (8), but a similar endoscopic appearance can arise from different underlying cell programs, and routine histology does not report which signaling pathways are active. Cross-sectional imaging, including CT and MR enterography, captures transmural and extramural disease that endoscopy cannot reach, which is particularly important in Crohn’s disease, yet it reports structure rather than the immune or stromal circuits that drive it (27). Bulk transcriptomics, the most molecular of the standard approaches, averages signal across epithelium, immune cells, stroma, and vasculature, so a dominant compartment can mask or imitate the contribution of others.

This averaging matters for drug selection. A raised cytokine transcript in a bulk sample does not reveal which cells produce the ligand, which cells carry the receptor, or whether the relevant circuit sits in a tissue compartment a drug can reach. Two patients with comparable calprotectin and comparable endoscopic scores may carry different dominant niches, which plausibly contributes to their divergent responses to the same agent. Conventional biomarkers are good measures of how inflamed a patient is and poor measures of why, and mechanism is what treatment selection requires (28, 29).

Bulk and single-cell studies have begun to define molecular endotypes of IBD, including the recognition that resistance to therapy can track with particular cellular programs rather than with clinical phenotype (30, 31). Endotype thinking is a real advance, but a dissociated or averaged signal still discards where cells sit and which neighbors they engage. We use the term *spatial pathotype* to describe a disease state defined not only by cellular composition but also by anatomical location, cell–cell interactions, cytokine and metabolite gradients, extracellular matrix remodeling, lesion architecture, and prior treatment exposure. A *spatial pathotype* is a property of organized tissue rather than of a cell list (**Figure 1**). Spatial context therefore matters for pathogenesis: the functional consequences of a given fibroblast state or cytokine pathway are not uniform across the intestinal mucosa but are shaped by the surrounding cellular neighborhood. Conceptualizing IBD as a mosaic of spatially defined pathotypes provides a translational framework that more closely reflects how therapies exert their effects, namely through modulation of specific cellular interactions and signaling networks within discrete tissue niches.

**Figure 1:**
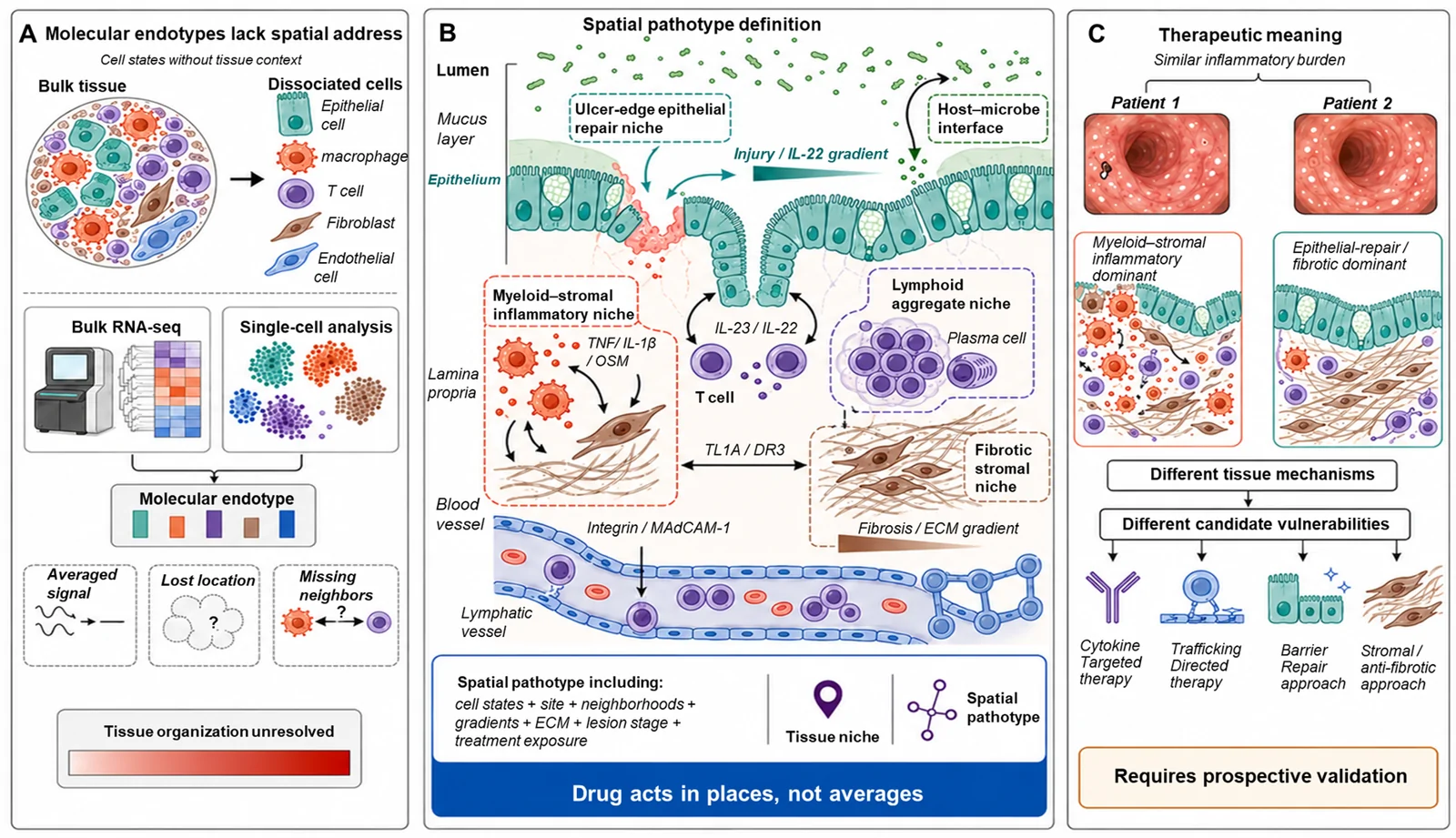
From molecular endotypes to spatial pathotypes in inflammatory bowel disease. **(A)** Bulk profiling identifies averaged molecular programs, whereas dissociated single-cell analysis resolves cell states; both incompletely preserve tissue position, cellular neighborhoods, and signaling gradients. **(B)** A *spatial pathotype* is proposed here as an operational tissue-level framework integrating anatomical site, cellular composition, cell–cell interactions, signaling gradients, extracellular-matrix remodeling, lesion stage, and treatment exposure. **(C)** Distinct *spatial pathotypes* may nominate different candidate therapeutic strategies, even among patients with comparable inflammatory burden, but these relationships require prospective, treatment-specific validation.

The central question of this review is therefore how spatial multi-omics can convert mucosal disease niches into therapeutic targets, biomarkers, pharmacodynamic assays, and precision clinical trials in Crohn’s disease and ulcerative colitis. Several reviews have summarized single-cell and spatial methods, and others have cataloged IBD cell atlases (32–34); the emphasis here is instead on how mucosal immune niches can guide drug development, pharmacodynamic assessment, biomarker qualification, and biomarker-enriched trial design in both diseases. Each niche is considered as a potential source of targets, a readout of drug action, and a basis for patient stratification, and that perspective is carried consistently from discovery science through trial design. To address this question, the review first defines the spatial organization of healthy and diseased mucosa, then considers single-cell and spatial technologies as translational tools, and finally examines how niche-level information may support target discovery, biomarker development, and trial design. Evidence from microbiome studies, spatial technologies, and therapeutic development is integrated where it informs the biological and translational significance of mucosal immune niches, with particular emphasis on how these niches shape disease heterogeneity, therapeutic vulnerability, and biomarker development, and with conclusions guided by the robustness of the available evidence.

### 1.1 Literature search and review framework

This article is a narrative review rather than a systematic review or meta-analysis and is intended to provide a critical, translational synthesis of the field rather than an exhaustive or formally weighted evaluation of all available evidence. Relevant publications were identified through searches of PubMed, Web of Science, and Scopus, while ongoing and completed clinical trials were identified through ClinicalTrials.gov. The reference lists of key articles were also screened to identify additional relevant studies. Searches primarily covered January 2014 to August 29, 2026, with earlier seminal studies included where necessary to describe foundational technologies, established therapeutic pathways, or key clinical evidence. Search terms combined inflammatory bowel disease, Crohn’s disease, and ulcerative colitis with mucosal immunology, single-cell RNA sequencing, single-nucleus RNA sequencing, spatial transcriptomics, spatial proteomics, multiplex imaging, spatial metabolomics, mucosal niche, fibrostenosis, fistula, postoperative recurrence, anti-TNF response, IL-23, OSM, TL1A, JAK–STAT, integrins, S1P, biomarker qualification, pharmacodynamic endpoint, and adaptive trial design. Priority was given to human tissue studies, single-cell and spatial atlases, translational biomarker studies, clinical trials, and consensus statements. Animal and *in vitro* studies were included when they clarified mechanistic plausibility but were interpreted as supportive rather than clinically validating evidence.

## 2 Spatial organization of the intestinal mucosa in health, Crohn’s disease, and ulcerative colitis

### 2.1 Normal mucosal architecture as the reference map

Disease niches can only be read against the organization of healthy tissue, and healthy intestinal mucosa is spatially patterned rather than randomly arranged. Along the crypt-surface axis, intestinal stem cells at the crypt base give rise to transit-amplifying progenitors that differentiate as they migrate upward into absorptive and secretory lineages, the latter including goblet cells, enteroendocrine cells, tuft cells, and, in the small intestine, Paneth cells that occupy the crypt base alongside stem cells (35, 36). Single-cell surveys of small intestine and colon, and integrated atlases spanning developmental time and anatomical region, show that epithelial identity, immune composition, and stromal programs all vary by position and segment, so ileum, colon, and rectum are distinct tissue contexts rather than interchangeable samples (35–37).

Beneath the epithelium, the lamina propria holds a structured mix of resident macrophages, dendritic cells, T and B lymphocytes, plasma cells, and innate lymphoid cells, organized around fibroblasts, endothelial cells, lymphatics, pericytes, and enteric neurons within an extracellular matrix scaffold (38, 39). Stromal cells are not passive filler; defined fibroblast subsets occupy specific positions, support epithelial stem cell maintenance, and shape immune localization through chemokines and matrix (38). Microbial communities are themselves spatially organized, separated from the epithelium by mucus in the colon and engaging the surface differently along the gut, so mucosa-associated microbes and their metabolites form local interfaces distinct from the luminal or fecal community (39). Homeostasis is therefore a property of arrangement, and disease can be read as the rearrangement of these compartments.

### 2.2 Spatial disruption in Crohn’s disease versus ulcerative colitis

Crohn’s disease and ulcerative colitis disrupt this architecture in characteristically different ways, though the contrast should not be drawn too simply. Ulcerative colitis is typically continuous and confined to the mucosa, beginning in the rectum and extending proximally, with epithelial erosion, goblet cell depletion, crypt distortion, neutrophil-rich cryptitis, and a plasma cell-rich lamina propria (7). Crohn’s disease is more often discontinuous and transmural, frequently involves the ileum, and is complicated over time by strictures, fistulae, and postoperative recurrence (6). Single-cell and spatial studies show that the two diseases share inflammatory modules, including overlapping cytokine and stromal programs, but distribute them differently across tissue depth and segment (30, 31, 40) (**Table 1**).

**Table 1:** Spatial organization of the intestinal mucosa in health, ulcerative colitis, and Crohn’s disease: shared and disease-distinct features with translational implications.

Spatial dimension	Reference architecture in health	Ulcerative colitis (UC)	Crohn’s disease (CD)	Shared vs. disease-distinct pattern and translational implication	References
*Tissue-level architecture*					
Segmental distribution and extent	Epithelial, immune, and stromal composition varies along the ileum–colon–rectum axis; intestinal segments are non-interchangeable tissue contexts.	Continuous colonic inflammation, usually beginning in the rectum and extending proximally.	Discontinuous lesions with frequent ileal involvement; any gastrointestinal segment may be affected.	**Distinct —** Segment and location should be annotated; findings from colonic UC should not be extrapolated directly to ileal CD.	(6, 7, 35–37)
Depth of tissue involvement	The crypt–surface axis organizes the mucosa, whereas vessels, lymphatics, nerves, smooth muscle, and matrix extend through deeper layers.	Predominantly mucosal inflammation.	Transmural inflammation involving the submucosa, muscularis, and adjacent mesentery.	**Distinct —** Mucosal biopsy samples the dominant inflammatory compartment in UC but incompletely represents transmural CD; deeper tissue and imaging are needed for complication-focused studies.	(6, 7, 35, 36)
*Epithelial compartment*					
Secretory lineages and mucus barrier	Goblet, enteroendocrine, and tuft cells arise along the crypt–surface axis; Paneth cells occupy the small-intestinal crypt base, and mucus limits microbial contact with the colonic epithelium.	Goblet-cell depletion, crypt distortion, and surface epithelial erosion.	Paneth-cell dysfunction in ileal disease, with impaired antimicrobial-peptide output; barrier injury also occurs across affected segments.	**Partly shared and site dependent —** Mucus loss and antimicrobial dysfunction alter host–microbe contact and provide complementary readouts of barrier injury and repair.	(7, 35, 36, 39)
Injury, repair, and metaplastic states	Continuous epithelial renewal restores mature crypt–surface architecture after injury.	Wound-associated, regenerative, and metaplastic states occur at ulcer edges and regenerating crypts.	Regenerative and metaplastic programs include specialized CD-associated epithelial states.	**Shared injury–repair axis with context-specific states —** Persistent regenerative or metaplastic programs may indicate incomplete molecular healing despite reduced inflammation.	(35, 36, 41, 42)
*Immune and stromal compartments*					
Immune organization and inflammatory modules	Resident macrophages, dendritic cells, lymphocytes, plasma cells, and innate lymphoid cells are spatially organized around stromal and vascular structures.	Plasma-cell-rich lamina propria and neutrophil-rich cryptitis, with extensive epithelial–immune rewiring.	Inflammatory mononuclear phagocytes, fibroblasts, and lymphocytes form pathogenic modules; myeloid heterogeneity varies by lesion and tissue depth.	**Shared modules, distinct organization —** Similar cytokine programs are distributed differently across segment and tissue depth; spatial configuration may be more informative than bulk abundance alone.	(7, 30, 31, 38–40)
Stromal and extracellular-matrix architecture	Positionally distinct fibroblast subsets support epithelial maintenance and organize immune localization through chemokines and matrix.	Inflammatory and remodeling fibroblast states reshape the colonic mesenchyme.	Inflammatory, matrix-producing, and remodeling fibroblast states extend across deeper layers and are prominent in fibrostenotic disease.	**Shared cell states, greater transmural remodeling in CD —** Stromal programs may explain incomplete tissue healing and provide candidate pharmacodynamic readouts, although their causal role remains incompletely defined.	(30, 38, 40, 46)
*Complication-specific niches and contextual modifiers*					
Stricturing behavior	—	Not characteristic; fixed fibrostenosis is uncommon.	Myofibroblast-dominated niches couple stromal cells, smooth muscle, immune cells, and extracellular matrix across tissue layers.	**CD-predominant —** Deep tissue or imaging is required; distinguishing inflammatory, mixed, and fixed fibrosis is central to trial enrichment and endpoint selection.	(6, 44, 46, 47)
Fistulizing behavior	—	Not characteristic.	Epithelialized tracts contain distinct fibroblast layers, inflammatory infiltrates, epithelial-to-mesenchymal transition-like programs, and microbial proximity.	**CD-predominant —** These maps define candidate tissue readouts for local or stromal-directed interventions, but predictive and pharmacodynamic utility remains unvalidated.	(6, 45, 116, 117)
Residual molecular disease and postoperative recurrence	—	Epithelial, plasma-cell, macrophage, or fibroblast programs may persist after apparent mucosal healing.	Residual programs may persist after healing; postoperative recurrence provides a predictable site and timing for early reactivation.	**Shared residual disease; postoperative recurrence is CD-focused —** Longitudinal sampling can test whether niche persistence predicts relapse or treatment response.	(43, 111, 118, 149)
Contextual modifier axes	Age and anatomical region shape baseline epithelial, immune, and stromal composition.	Disease extent, age at onset, and treatment exposure alter tissue states.	Ileal versus colonic location, disease behavior, age at onset, surgery, and prior therapy add major axes of heterogeneity.	**Shared modifiers —** Site, disease behavior, age, and treatment history should be prespecified and reported; paired inflamed/non-inflamed sampling improves interpretability.	(37, 43–49)

The listed patterns summarize common tissue contexts rather than absolute diagnostic boundaries. Most spatial evidence derives from cross-sectional human cohorts and therefore establishes association rather than causality. —, not applicable. CD, Crohn’s disease; UC, ulcerative colitis. Bold text in the ‘Shared vs. disease-distinct pattern and translational implication’ column indicates the qualitative classification of each spatial feature as shared, disease-distinct, or context/site dependent between UC and CD.

The shared-versus-distinct pattern has therapeutic meaning. Convergent inflammatory circuits may explain why several drug classes work in both diseases, while differences in anatomical depth and stromal involvement may explain why transmural and fibrostenotic complications are far more prominent in Crohn’s disease and why luminal-mucosal endpoints capture Crohn’s biology incompletely. Spatial profiling helps separate a mechanism that is genuinely disease-specific from one that is shared but positioned differently, a distinction that bulk comparison tends to blur.

### 2.3 Disease-relevant regions beyond the most inflamed biopsy

A practical consequence of spatial thinking is that the most inflamed biopsy is not always the most informative. Mechanistic and predictive signals may arise at the ulcer edge, in regenerating crypt zones, in endoscopically normal but molecularly active mucosa, in fibrotic strictures, in fistula-associated tissue, at postoperative recurrence sites, or in histologically healed mucosa that still harbors residual programs (41–43). Studies of metaplastic and specialized epithelial states associated with inflammation, and of fibrosis- and fistula-associated stromal niches, suggest that therapeutically relevant biology can reside outside the zone of maximal inflammation (41, 42, 44, 45).

This perspective positions tissue sampling as a critical component of study design rather than a downstream technical consideration. Because biologically informative signals may be distributed across inflammatory cores, lesion boundaries, repair-associated regions, and macroscopically quiescent tissues, sampling strategies that capture spatial heterogeneity are essential for accurately defining disease-associated niches. Accordingly, paired sampling of inflamed and non-inflamed regions should be strongly considered in studies aiming to identify residual molecular programs, complication-associated tissue states, or candidate biomarkers of therapeutic response.

### 2.4 Spatial heterogeneity by disease location and behavior

Tissue context in IBD is layered. Ileal and colonic Crohn’s disease differ in baseline epithelial and immune composition; ulcerative colitis and rectal disease differ again; and stricturing and fistulizing behaviors add tissue programs largely absent from uncomplicated mucosal inflammation (44–47). Postoperative recurrence offers a tractable window onto early reactivation, since the timing and location of new inflammation can be anticipated, and recent spatial work across anatomical locations in this setting shows how inflammatory signatures differ even within a single patient’s reconnected bowel (43). Pediatric-onset disease and treatment-naive versus treatment-exposed mucosa add further axes, because prior drug exposure reshapes the very niches a study aims to read, and molecular features can even predict extension of limited disease (48, 49).

### 2.5 How far do niche definitions generalize?

That healthy and diseased mucosa are spatially organized, and that Crohn’s disease and ulcerative colitis distribute shared inflammatory programs differently across tissue depth and bowel segment, is now well established (30, 31, 38, 40). The harder question is reproducibility. Most spatial maps still come from modest, single-center cohorts, and it remains unclear how reliably a discrete niche definition survives a change of platform, population, or disease context. This matters because a spatial finding carries an address: a niche characterized in colonic ulcerative colitis cannot be assumed to behave identically in ileal or fibrostenotic Crohn’s disease, and treating it as portable without testing risks importing a cohort-specific artifact. Closing this gap depends less on new technology than on disciplined sampling, namely paired inflamed and non-inflamed biopsies annotated for site, depth, and treatment history and replicated across centers, which is what would let niche definitions generalize rather than remain local descriptions.

## 3 Technologies for mapping IBD mucosal niches: from single-cell omics to spatial multi-omics

### 3.1 Single-cell and single-nucleus omics as the cellular foundation

Spatial interpretation depends on a high-quality reference of cell states, and single-cell methods supply it (**Figure 2**). Droplet-based single-cell RNA sequencing made it feasible to profile tens of thousands of cells per sample (50, 51), and the same chemistry underpins the IBD atlases that first resolved disease-associated epithelial, immune, and stromal states (30, 31). Single-nucleus RNA sequencing can facilitate profiling of frozen or difficult-to-dissociate specimens, including fibrotic and surgical tissues, although nuclear RNA profiles differ systematically from whole-cell transcriptomes. Multimodal variants add layers: CITE-seq measures surface proteins alongside transcripts (52), single-cell ATAC-seq reports chromatin accessibility (53), and combined assays such as SHARE-seq link the two within a cell (54).

**Figure 2:**
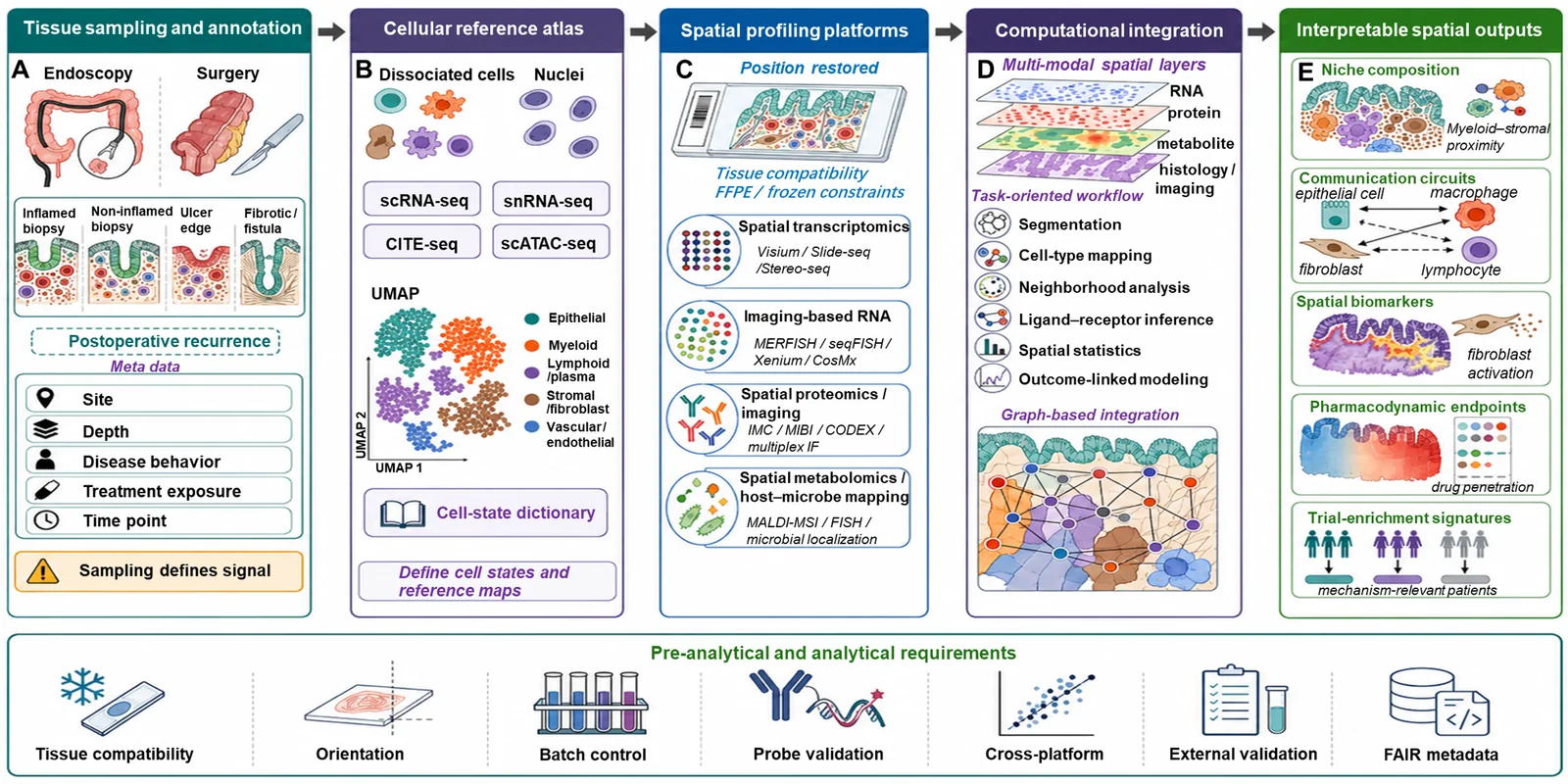
Integrated workflow for spatial multi-omics mapping of mucosal niches in inflammatory bowel disease. **(A)** Standardized sampling captures inflamed, macroscopically non-inflamed, and complication-specific tissues together with anatomical site, tissue depth, treatment exposure, and sampling time point. **(B)** Single-cell and single-nucleus assays provide a reference of cell states and molecular programs. **(C)** Spatial transcriptomic, proteomic, imaging, metabolomic, and host–microbe approaches map these states within tissue. **(D)** Multimodal integration supports cell-type mapping, neighborhood analysis, ligand–receptor inference, spatial statistics, and outcome-linked modeling. **(E)** Interpretable outputs include niche maps, candidate communication circuits, spatial signatures, pharmacodynamic readouts, and trial-enrichment markers. Cross-cutting quality requirements include tissue compatibility, standardized orientation, batch and probe controls, cross-platform benchmarking, harmonized metadata, and external validation.

These methods define what cell states exist and which programs they run, but they pay a price for resolution. Dissociation removes cells from their tissue coordinates and can bias recovery against fragile or matrix-embedded populations, so a dissociated atlas underrepresents exactly the stromal and epithelial-edge cells that spatial work later shows to be important. Computational integration tools, including Harmony, Seurat anchor-based integration, Scanpy workflows, and probabilistic models such as scVI, mitigate batch effects and allow atlases to be combined, but they cannot restore position (55–58). Single-cell data are therefore the necessary cellular dictionary, not the map.

### 3.2 Spatial transcriptomics platforms and trade-offs

Spatial transcriptomics restores position, and platform choice is a trade-off between coverage, resolution, and clinical practicality (**Table 2**). Sequencing-based, array-capture methods such as the original Spatial Transcriptomics chemistry and its successors provide near-whole-transcriptome breadth, with progressively finer resolution from the original spot size toward near-cellular grids; bead- and chip-based approaches including Slide-seq, high-definition arrays, and Stereo-seq push resolution further (59–62). The cost is that capture-based data often mix several cells per unit and require computational deconvolution to assign signal to cell types. Imaging-based, targeted methods take the opposite approach: MERFISH, seqFISH, and commercial platforms such as CosMx and Xenium resolve single cells or subcellular transcripts but read a predefined gene panel rather than the whole transcriptome (63–67). Probe-based profiling such as the GeoMx system supports region-of-interest analysis with high multiplexing in archival tissue (68).

**Table 2:** Technologies for mapping IBD mucosal niches: analytical capabilities and relative translational scalability.

Modality/representative methods	Resolution & molecular readout	Main translational feature/limitation	Relative cost	Throughput/turnaround	Automation/expertise	Overall scalability	References
*Single-cell foundation — defines cell states but does not preserve tissue coordinates*							
scRNA-seq/snRNA-seq — droplet-based scRNA-seq; snRNA-seq	Single cell/nucleus; whole-transcriptome RNA	Defines reference cell states; loses tissue coordinates and may introduce dissociation or recovery bias	High	High/Moderate	Moderate/High	Moderate	(50, 51)
Multimodal single-cell profiling — CITE-seq; scATAC-seq; SHARE-seq	Single cell; protein, chromatin, or paired RNA–chromatin	Adds regulatory or protein layers but increases experimental and integration complexity	Very high	Moderate/Long	Low/Very high	Low	(52–54)
*Spatial transcriptomics — sequencing-based capture platforms*							
Capture-based spatial transcriptomics — ST; Visium and related platforms	Spot-level to near-cellular; near-whole-transcriptome RNA	Broad discovery with tissue context; deconvolution often required and FFPE compatibility is platform dependent	High	Moderate/Moderate	Moderate/High	Moderate	(59, 69–73)
High-resolution spatial arrays — Slide-seq/Slide-seqV2; HDST; Stereo-seq	~2–10 μm or smaller bins; genome-wide RNA	High spatial granularity but specialized workflows; nominal resolution may exceed effective biological resolution	Very high	Moderate/Long	Low/Very high	Low	(60–62)
*Spatial transcriptomics — imaging-based targeted platforms*							
Multiplexed single-molecule FISH — MERFISH; seqFISH+	Single-cell/subcellular; targeted RNA panels	Sensitive localization but predefined panels and imaging complexity constrain cohort scale	Very high	Low/Long	Low/Very high	Low	(63–65)
Commercial imaging platforms — CosMx SMI; Xenium	Single-cell/subcellular; targeted RNA ± protein	FFPE-compatible and suitable for archival/trial tissue; segmentation and platform-specific workflows affect portability	Very high	Moderate/Moderate	High/High	Moderate	(66, 67, 72, 73)
ROI profiling — GeoMx Digital Spatial Profiler	Region/ROI-level; high-plex RNA and protein	FFPE-compatible and relatively scalable; ROI selection may obscure cellular heterogeneity	High	High/Moderate	High/High	High	(68)
*Beyond transcripts — protein, metabolite, microbial, and image-derived layers.*							
Spatial proteomics/multiplex imaging — IMC; MIBI; CODEX; multiplex IF	Single-cell/subcellular; dozens of proteins in situ	Directly localizes therapeutic proteins; antibody validation and quantitative reproducibility remain limiting	High	Moderate/Moderate	Moderate/High	Moderate	(74–78)
Spatial metabolomics/pharmacology — MALDI-MSI	Tissue region to near-cellular; metabolites and drug distribution	Maps metabolic gradients and local drug exposure; specialized identification and analysis required	High	Moderate/Moderate	Moderate/Very high	Moderate	(79, 80)
Host–microbe spatial profiling — multiplex microbial imaging; integrative profiling	Single microbe to tissue region; microbial–host localization	Resolves local host–microbe interfaces; standardization and causal interpretation remain early	High	Low/Long	Low/Very high	Low	(81–83)
Digital pathology — deep learning on whole-slide images	Whole-slide morphology; image-derived features	Uses routine FFPE slides and may provide scalable surrogates; models require interpretability and external validation	Low	High/Short	High/High	High	(84–86)
*Computational analysis, integration, and validation*							
Atlas integration/batch correction — Harmony; Seurat; Scanpy; scVI	Computational layer; integrated cell-state atlases	Supports cross-dataset integration; over-correction may obscure true biological variation	Low	High/Short	High/High	High	(55–58)
Deconvolution/segmentation — cell2location; RCTD; Tangram; Baysor	Input-dependent; cell-type assignment and boundaries	Essential for interpreting spots and imaging data; depends on reference and segmentation quality	Low	High/Short	High/High	High	(87–90)
Neighborhood/ligand–receptor analysis — Squidpy; Giotto; NicheNet; CellChat; CellPhoneDB	Computational layer; neighborhoods and inferred communication	Prioritizes candidate interactions but does not establish active signaling or causality	Low	High/Short	High/High	High	(91–95)
Benchmarking/outcome modeling — SpatialData; standardized metrics; multi-omics ML	Analysis/evaluation layer; harmonized data and predictive models	Supports reproducibility and outcome modeling; external validation and interpretability remain essential	Moderate	High/Moderate	Moderate/Very high	Moderate	(96–99)

Relative implementation characteristics are qualitative assessments for comparison within this review rather than fixed platform specifications or monetary estimates. Values may vary with specimen type, workflow, study scale, and local resources; paired ratings follow the order shown in the column headings. Overall scalability reflects feasibility for larger multicenter or clinical-trial cohorts, and spatial resolution refers to nominal rather than effective single-cell resolution. CITE-seq, cellular indexing of transcriptomes and epitopes by sequencing; CODEX, co-detection by indexing; FFPE, formalin-fixed paraffin-embedded; FISH, fluorescence in situ hybridization; HDST, high-definition spatial transcriptomics; IBD, inflammatory bowel disease; IMC, imaging mass cytometry; MALDI, matrix-assisted laser desorption/ionization; MERFISH, multiplexed error-robust fluorescence in situ hybridization; MIBI, multiplexed ion beam imaging; MSI, mass spectrometry imaging; ROI, region of interest; RCTD, robust cell type decomposition; scATAC-seq, single-cell assay for transposase-accessible chromatin sequencing; scRNA-seq, single-cell RNA sequencing; SMI, spatial molecular imaging; snRNA-seq, single-nucleus RNA sequencing; ST, spatial transcriptomics.

For IBD, three practical criteria usually dominate. Compatibility with formalin-fixed, paraffin-embedded tissue determines whether large archival and trial cohorts can be used at all. Single-cell resolution determines whether epithelial-edge, neutrophil, and fibroblast neighborhoods can be told apart. Panel size and cost determine feasibility at the scale a multicenter study or trial requires (69–71). Recent systematic comparisons of sequencing- and imaging-based platforms make clear that no single technology is best for every question, and that the biological target, whether epithelial repair, immune neighborhood structure, fibroblast state, or host-microbe contact, should drive the choice (32, 72, 73).

### 3.3 Spatial proteomics and multiplex imaging

Protein-level spatial methods sit closer to drug action than RNA, because receptors, ligands, and signaling intermediates are what most therapies engage. Imaging mass cytometry and multiplexed ion beam imaging use metal-tagged antibodies to quantify dozens of proteins *in situ*, while fluorescence-based approaches such as CODEX and multiplex immunofluorescence reach comparable depth with accessible instrumentation (74–77). Combined RNA-protein detection allows transcript and antigen to be read in the same section. Applied to ulcerative colitis, spatial proteomic atlases have resolved inflammatory cell neighborhoods and reported features associated with resistance to TNF inhibition, which shows how protein-level maps can move toward companion-diagnostic-style readouts (78).

The limitations are specific and should temper interpretation. Antibody validation, panel size, autofluorescence, and quantification reproducibility all constrain these assays, and receptor presence on an imaged section indicates localization rather than active signaling or target engagement. Spatial proteomics is therefore most powerful when used to validate, at the protein level and in the right tissue compartment, hypotheses generated by transcriptomic mapping.

### 3.4 Spatial metabolomics, microbiota-adjacent signals, and digital pathology

Two further layers extend the picture beyond cells and transcripts. Mass spectrometry imaging, principally MALDI-based approaches, maps lipids, bile acids, amino acid metabolites, and, in some settings, drug distribution across tissue, and has begun to reveal region-specific metabolic signatures of bowel inflammation (79). Integrated with host transcriptomics, these gradients can generate metabolite-linked therapeutic hypotheses and provide spatial information on local drug exposure (80). Host–microbe spatial profiling, using high-resolution imaging or capture-based approaches, locates mucosa-associated microbial communities relative to mucus, epithelium, crypts, and inflammatory cell aggregates, capturing local interfaces that are poorly represented by fecal sequencing (81–83). Local microbial activity may also generate gradients of short-chain fatty acids, bile acids, and tryptophan-derived metabolites that influence adjacent epithelial, myeloid, lymphoid, and stromal programs. Integrating microbial imaging or spatial microbial profiling with host transcriptomics, proteomics, and spatial metabolomics could therefore identify host–microbe co-localization patterns relevant to barrier dysfunction and inflammatory niche formation (39, 81–83). However, spatial co-localization remains associative and does not by itself establish microbial causality, which requires longitudinal and functional validation.

Digital pathology adds a pragmatic layer. Whole-slide images are already generated in routine practice, and weakly supervised deep learning can extract disease-relevant features from them, which raises the possibility that some spatial discoveries could eventually be approximated by inexpensive, scalable image analysis rather than full multi-omics (84–86). That prospect remains to be validated, but it is the kind of derivative assay most likely to reach clinical workflows.

### 3.5 AI-assisted multimodal integration

Spatial data are large, noisy, and modality-specific, so computation is not an optional add-on. Deconvolution methods such as cell2location, RCTD, and Tangram assign cell types to capture-based spots; segmentation tools such as Baysor delineate cells in imaging data; analysis frameworks including Squidpy and Giotto detect neighborhoods and spatial statistics; and ligand–receptor tools such as NicheNet, CellChat, and CellPhoneDB infer candidate communication (87–95). Standard data structures and benchmarking efforts are beginning to make these pipelines comparable across studies (96, 97). More recent machine-learning architectures extend these analyses toward multimodal representation and prediction. Graph neural networks, including graph-attention architectures, can represent cells or spatial spots as nodes linked by spatial proximity or learned molecular similarity, supporting spatial-domain detection and multicellular-neighborhood modeling. Transformer-based models offer a complementary strategy for capturing longer-range tissue relationships and integrating spatial molecular profiles with histology and other modalities. In IBD, however, their use for treatment-response or outcome prediction remains exploratory, with available spatial cohorts generally limited in size and heterogeneous in sampling, platform, and clinical annotation. This raises concerns about overfitting, center-specific domain shift, interpretability, and external transportability. Externally validated and explainable spatial predictors therefore remain limited, and prospective multicenter validation will be required before such models can support clinical decision-making (98).

### 3.6 Choosing a platform and reading its output

Each modality has a core capability that is not in dispute: single-cell methods define cell states, spatial transcriptomics restores position, spatial proteomics reads protein-level circuits, and mass spectrometry imaging adds metabolite and drug distribution. Where the picture is less settled is in cross-platform concordance and in how several assays actually perform on the formalin-fixed, paraffin-embedded tissue that trials and archives provide, since benchmarking is recent and agreement between platforms has been uneven (72, 73, 99). Two cautions follow for anyone using these tools translationally. Platform choice should track the biological question and the constraints of the available tissue rather than the novelty of a method. Computational output should be read as a prioritized list of testable claims rather than as a finding in itself, because ligand–receptor scoring proposes communication without proving it and neighborhood models built on small cohorts overfit readily (96, 97, 99). The field would benefit from harmonized benchmarking, shared reference data structures, and external testing of models that link spatial features to outcomes.

## 4 Disease-defining mucosal niches and cellular circuits in IBD

The major mucosal niches are best read as functionally relevant tissue units with potential therapeutic significance. For each, we describe its cellular composition and biological functions, draw out its implications for pathogenesis and therapeutic targeting, and weigh the strength and limitations of the current evidence.

### 4.1 Epithelial injury, incomplete repair, and metaplastic niches

The epithelium is an active regulator of inflammation and repair, not merely its victim. Chronic injury disturbs the crypt–surface program. Stem and transit-amplifying compartments are remodeled, goblet cells and their mucus are depleted, and Paneth cell abnormalities in ileal Crohn’s disease compromise antimicrobial peptide output (36). These are immunological events as much as structural ones. Loss of the mucus barrier and of α-defensin and lysozyme-rich Paneth cell secretions alters which microbes contact the epithelium, lowering the threshold for innate activation and shaping the cytokine milieu that recruits myeloid and lymphoid cells to the lesion. Epithelial cells in turn read and instruct this milieu. They respond to IL-22 from group 3 innate lymphoid cells and T cells through mucin and antimicrobial-peptide programs, interact with TNF and OSM-driven stromal intermediaries, and participate in IL-23-associated circuits that several therapies target. Single-cell and spatial studies have identified wound-associated and regenerative epithelial states, and metaplastic programs resembling pyloric or Brunner’s-gland epithelium, that mark attempted repair but can also signal persistent injury (41, 42). A specialized epithelial cell type specifically associated with Crohn’s disease has been described and characterized multimodally, and metaplasia has been integrated across inflammatory gut conditions, which places these states within a broader injury-repair landscape rather than treating them as isolated curiosities (41, 42). A recurring theme is incomplete repair: epithelium can adopt a regenerative or fetal-like program that closes a wound transiently without restoring mature, functional architecture, leaving a fragile surface prone to re-injury and to continued immune stimulation.

For drug development, the value of this niche is twofold. Epithelial states can serve as damage sensors and as candidate readouts of repair that are distinct from the suppression of inflammatory transcripts, which means a treatment could quiet inflammation while leaving epithelial architecture unrestored. They also nominate barrier-restoring strategies as a therapeutic axis separate from immune suppression (100, 101). The caution is that pathways promoting epithelial regeneration intersect with dysplasia and cancer biology, so any repair-directed approach carries a safety question that spatial localization can help frame but not answer.

### 4.2 Myeloid, neutrophil, and antigen-presenting-cell niches

Myeloid cells organize much of the inflammatory tissue in IBD, and their ontogeny matters as much as their number. Most adult lamina propria macrophages are continuously replenished from circulating monocytes, although long-lived tissue-resident subsets coexist. During active IBD, increased recruitment and incomplete maturation of monocyte-derived macrophages contribute to the accumulation of inflammatory myeloid states. Dendritic cells shape the adaptive response through antigen presentation and IL-12/IL-23 production, and neutrophils accumulate at sites of active epithelial breach. Spatial analysis at single-cell resolution has resolved this macrophage and neutrophil heterogeneity and the geographic relationships between these populations in human IBD tissue (40). Granulocyte-rich regions cluster at sites of ulceration and cryptitis, and inflammatory monocyte-derived macrophages sit close to fibroblasts in arrangements that sustain local cytokine production. These neighborhoods are the principal tissue sources of TNF, IL-1β, IL-23, OSM, and chemokines, and their cellular composition has been linked to therapeutic outcome (102, 103).

The clearest worked example is the IL-1-driven stromal–neutrophil circuit, in which myeloid-derived IL-1β activates fibroblasts to produce neutrophil-recruiting chemokines and colony-stimulating factors, establishing a self-amplifying inflammatory program. This circuit was defined by integrating bulk and single-cell transcriptomics, quantitative histopathology, and *in situ* localization across three IBD cohorts (n = 376), with associated neutrophil and fibroblast signatures subsequently linked to non-response across four additional cohorts (n = 343) receiving anti-TNF, corticosteroid, or anti-integrin therapy (102). OSM defines a parallel axis through OSMR signaling on stromal and endothelial cells rather than directly on leukocytes. OSM is produced predominantly by inflammatory myeloid and other hematopoietic cells and acts on OSMR-expressing stromal populations, thereby amplifying a myeloid–stromal inflammatory circuit (103, 104). The original OSM analysis evaluated this association across five human IBD datasets (n = 227), including infliximab- and golimumab-treated cohorts, providing cross-dataset support for its reproducibility (103). A single-cell-defined pathogenic cellular module comprising inflammatory mononuclear phagocytes, activated stromal cells, activated T cells, and IgG plasma cells was subsequently detected across four independent ileal Crohn’s disease cohorts (n = 441); its presence at diagnosis was associated with failure to achieve durable corticosteroid-free remission during anti-TNF therapy (31). Across these cohorts, prior biologic exposure varied and was not consistently reported using harmonized definitions, which should be considered when comparing treatment-response-associated tissue signatures across studies.

The therapeutic reading of these neighborhoods is direct. Persistent myeloid-stromal inflammation is a plausible reason why some patients fail mechanistically appropriate drugs, and the producing and responding cells in these neighborhoods define what a niche-directed therapy must engage. The same neighborhoods also provide candidate predictive markers, discussed in Section 6, and candidate pharmacodynamic readouts. For example, contraction of a pathogenic myeloid cluster would provide a more tissue-specific signal of drug effect than a fall in a circulating inflammatory marker.

### 4.3 Lymphoid niches: T cells, B cells, plasma cells, ILCs, and tertiary lymphoid structures

The adaptive and innate lymphoid compartments are spatially organized. Effector T cells, regulatory T cells, tissue-resident memory cells, and cytotoxic states distribute differently by disease and segment. Th17 and Th1-like effector programs concentrate in actively inflamed mucosa, while regulatory T cells show a functional plasticity that can let them acquire effector features under sustained inflammation. Single-cell analysis of colonic CD8 T cells, intraepithelial T-cell heterogeneity in Crohn’s disease, and clonally related adaptive populations in ulcerative colitis have shown how these states expand and reorganize in disease (105–107). B cells, plasmablast-skewed plasma cell populations, and innate lymphoid cells contribute to chronicity and local antibody responses (31, 108, 109). Among them, group 3 innate lymphoid cells operate within the IL-23–IL-22 axis, sitting at the interface of innate cytokine signaling and epithelial defense and tying this compartment to the same IL-23 biology that several therapies target. Lymphoid aggregates and tertiary lymphoid structures act as organized immune hubs whose therapeutic meaning is still being defined. They may sustain local adaptive responses or, in some contexts, support regulated immunity, and their net effect in IBD remains unsettled.

Lymphoid architecture intersects directly with drug mechanism. Attrition of gut-associated lymphoid tissue tracks with response to anti-α4β7 integrin therapy in ulcerative colitis, linking a structural feature of tissue to the action of a trafficking-directed drug (110). B-cell expansion can interfere with the stroma-epithelium crosstalk required for mucosal healing, which connects a lymphoid program to repair rather than only to inflammation (111).

For drug mechanism, lymphoid organization could inform the use of anti-trafficking, cytokine-targeted, and immune-reprogramming strategies. These relationships are early, the role of tertiary lymphoid structures is genuinely uncertain, and the proposals need prospective testing. Even so, the link between gut-associated lymphoid tissue and anti-integrin response is among the clearer examples of a structural tissue feature that carries mechanistic, drug-specific information.

### 4.4 Stromal, vascular, lymphatic, and extracellular-matrix niches

Once viewed mainly as structural support, the stromal compartment is now recognized as an active organizer of IBD pathology. Fibroblasts comprise diverse inflammatory, matrix-producing, and remodeling states that occupy distinct anatomical positions and function as local signaling hubs. Activated subsets can amplify inflammation through OSM–OSMR and IL-6-family signaling, retain chemokines that guide leukocyte positioning, respond to TL1A/DR3 activation with combined inflammatory and pro-fibrotic programs, and promote matrix deposition under TGF-β control (38, 46). Remodeling of the colonic mesenchyme is a defining feature of disease, and inflammatory fibroblast states have repeatedly been associated with therapeutic resistance, partly because they maintain pathogenic myeloid–stromal circuits rather than simply accompanying inflammation (38, 102). Spatially resolved studies of colitis have further begun to define fibroblast trajectories and tissue distribution, suggesting that stromal activation follows coordinated anatomical patterns rather than occurring uniformly across the bowel (40).

Vascular and lymphatic networks likewise form biologically active microenvironments. Endothelial activation and angiogenesis influence leukocyte recruitment, tissue oxygenation, and local inflammatory persistence, while the endothelial surface provides the direct interface for anti-integrin and anti-MAdCAM-1 therapies. Impaired lymphatic drainage may prolong edema and delay the clearance of immune cells and antigens. Conversely, experimental enhancement of lymphatic function has been associated with reduced intestinal inflammation, identifying this compartment as a potentially relevant but still underexplored therapeutic target (112). Neurovascular and stromal organization is also shaped by enteric neurons and glia, which communicate with epithelial and immune cells to regulate motility, pain, barrier integrity, and fibrotic remodeling. Such interactions increasingly place neuroimmune signaling within the mucosal disease circuit rather than outside it (113).

Beyond providing structural support, the extracellular matrix actively regulates the behavior of surrounding cells. Matrix stiffness, cytokine sequestration, and chemokine presentation influence cell migration, activation, and signal propagation. Progressive matrix accumulation may also restrict drug diffusion into diseased tissue. Fibrotic lesions should therefore be regarded not simply as residual scar tissue, but as self-reinforcing microenvironments that preserve mechanical stress, inflammatory signaling, and impaired tissue repair.

Taken together, these observations help explain why inflammatory activity may decline while structural disease continues to progress. Current therapies suppress many immune pathways but incompletely address stromal activation, vascular remodeling, lymphatic dysfunction, and matrix organization. These compartments therefore provide both a biological explanation for incomplete healing and a largely underused source of therapeutic targets. Vascular and lymphatic niches are particularly relevant to existing trafficking-directed agents, whereas fibroblast and matrix programs may offer opportunities for antifibrotic treatment, tissue-directed pharmacodynamic assessment, and earlier identification of lesions at risk of irreversible remodeling.

### 4.5 Stricturing Crohn’s disease

Fibrostenosis is the clearest case where spatial methods add what bulk analysis cannot. Single-cell and high-resolution spatial studies have resolved fibroblast and myofibroblast heterogeneity, mapped fibrosis-associated networks onto tissue layers, and described a myofibroblast-dominated fibrotic niche that couples stromal cells, smooth muscle, immune cells, and matrix (44, 46, 47). Mechanistically, sustained TGF-β signaling drives the myofibroblast differentiation and collagen deposition that distinguish these niches. This program is reinforced by IL-6-family and TL1A/DR3 inputs and by a stiffening, cytokine-retaining matrix, which may explain why anti-inflammatory drugs that leave the TGF-β–matrix loop intact often fail to reverse established disease. These data begin to separate reversible inflammatory fibrosis from established structural remodeling. In practice strictures span a spectrum from predominantly inflammatory, through mixed inflammatory-fibrotic, to fixed fibrotic lesions, and these categories differ in their likely response to anti-inflammatory therapy. A practical limitation follows from anatomy: because the relevant fibrosis sits in the submucosa and muscularis, a mucosa-only biopsy can miss it, so niche assessment of stricturing disease will often require deeper or surgical tissue and alignment with cross-sectional imaging.

Here the reversible-versus-established distinction is what matters for treatment, because only the former is likely to respond to current anti-inflammatory drugs, and because patient selection for any future anti-fibrotic agent will depend on identifying the niche before scarring becomes fixed, an effort that has prompted consensus work to standardize stricture definitions and anti-fibrotic trial endpoints (101, 114, 115). No broadly validated anti-fibrotic therapy yet exists for Crohn’s strictures, so the immediate value of stricture niche maps is to define enrichment criteria and pharmacodynamic readouts for anti-fibrotic trials rather than to direct current care.

### 4.6 Fistulizing Crohn’s disease

Fistulae are better understood as structured niches than as simple inflammatory tunnels. Spatial and multimodal analysis have defined fibroblast niches that characterize Crohn’s fistulae and described the cellular mechanisms of perianal disease across diverse patients, revealing epithelialized tracts, distinct stromal layers, inflammatory infiltrate, and microbial proximity (45, 116, 117). A recurrent observation is an epithelial-to-mesenchymal transition-like fibroblast program and a specialized stromal compartment that organizes the tract.

From a development standpoint, fistula niche biology nominates stromal-reprogramming and locally delivered strategies, and it defines the cell populations a fistula-closing agent would need to affect. Because fistula tissue is rare and difficult to sample, the niche maps also argue for concentrating such tissue in standardized surgical biobanks and for aligning MRI, histopathology, and spatial omics on the same lesions, so that imaging-linked surrogate readouts could support trials without repeated deep sampling.

### 4.7 Postoperative recurrence and residual molecular disease after apparent healing

Postoperative recurrence supplies a longitudinal model of early reactivation, because the timing and location of new inflammation can be anticipated, and spatial signatures differ across anatomical locations within the same reconnected bowel (43). Separately, spatial and single-cell data suggest that mucosa which appears endoscopically or even histologically healed can retain residual molecular programs, including persistent fibroblast, plasma cell, macrophage, or epithelial-stress states (111, 118). Residual disease of this kind is a candidate explanation for relapse despite apparent response.

Together, these two settings define where a spatial pharmacodynamic endpoint would be most valuable. If residual niches predict relapse, then their persistence after treatment is a mechanistic early-warning signal, and their resolution is a more demanding and potentially more meaningful target than endoscopic appearance alone. Across all complication-specific niches, the common message is that luminal inflammation does not explain the tissue, and that anti-fibrotic, stromal-reprogramming, matrix-modifying, repair-oriented, or locally delivered strategies will require the niche-level resolution spatial multi-omics provides.

### 4.8 Association, causality, and the weight of surgical cohorts

Several niche-level phenomena are now reproducible enough to take seriously: inflammatory and matrix-producing fibroblast states in disease (38, 46), the myeloid-stromal and IL-1–stromal–neutrophil circuits associated with poor treatment response (102), an OSM-high stromal-immune module linked to anti-TNF non-response (103, 104), and the distinct fibroblast niches of stricturing and fistulizing Crohn’s disease (44–46). The recurring weakness is that these descriptions are overwhelmingly cross-sectional, so they establish association rather than causality, and they rarely tell us whether a niche drives disease, follows from it, or marks a stage still open to reversal. Compounding this limitation, many of the most detailed spatial atlases derive from surgical specimens and therefore preferentially represent severe, longstanding, treatment-refractory, fibrostenotic, or penetrating disease. This spectrum bias is particularly relevant to stricture and fistula atlases, for which deep or surgical tissue is often unavoidable, and signatures derived from such specimens should not be assumed to represent newly diagnosed, biologic-naïve, or medically controlled disease. The magnitude of this bias cannot yet be reliably quantified across published atlases because cohorts differ substantially in phenotype, anatomical site, prior treatment, specimen source, and profiling platform. More informative mitigation strategies include prospective endoscopic cohorts spanning disease severity and treatment history, paired inflamed and non-inflamed sampling, matched endoscopic and surgical specimens where feasible, and external validation of surgical-derived signatures in independent nonsurgical cohorts.

For now, such niches are best regarded as well-supported hypotheses about where to intervene and what to measure rather than as validated therapeutic targets. Longitudinal sampling can help distinguish disease-driving from stage-associated states: paired biopsies obtained before treatment and at predefined on-treatment time points can determine whether a niche is stable, contracts with effective therapy, or persists despite conventional clinical improvement. Such changes could then be evaluated as exploratory pharmacodynamic endpoints rather than inferred from cross-sectional associations alone. Establishing causal relevance will additionally require functional perturbation in appropriate model systems and replication across clinically diverse cohorts.

## 5 From spatial niches to drug target discovery and therapeutic development

### 5.1 A working framework for spatially actionable targets

Spatial multi-omics is most useful for drug development when it moves beyond descriptive mapping to prioritize testable and therapeutically tractable mechanisms (**Figure 3**). Here, we use the term spatially actionable target as a working concept for a molecule, pathway, cell state, or cell–cell interaction that satisfies several complementary considerations. The first is spatial enrichment, meaning reproducible concentration in a disease-relevant niche rather than diffuse expression. A candidate must also have an identified producing cell that generates the signal and an identified responding cell that carries the receptor or effector machinery. It must operate within a defined disease context of location, behavior, and treatment exposure. A feasible therapeutic modality must be able to reach the relevant compartment, and a measurable target-engagement or pharmacodynamic readout must be available. The safety concern must be assessable, including the consequences of inhibiting the pathway where it is protective. Finally, the level of evidence maturity should be stated. Operationally, these considerations can be grouped into seven domains: spatial enrichment, source–responder cell definition, disease context, therapeutic accessibility, measurable target engagement or pharmacodynamics, safety/selectivity, and evidence maturity.

**Figure 3:**
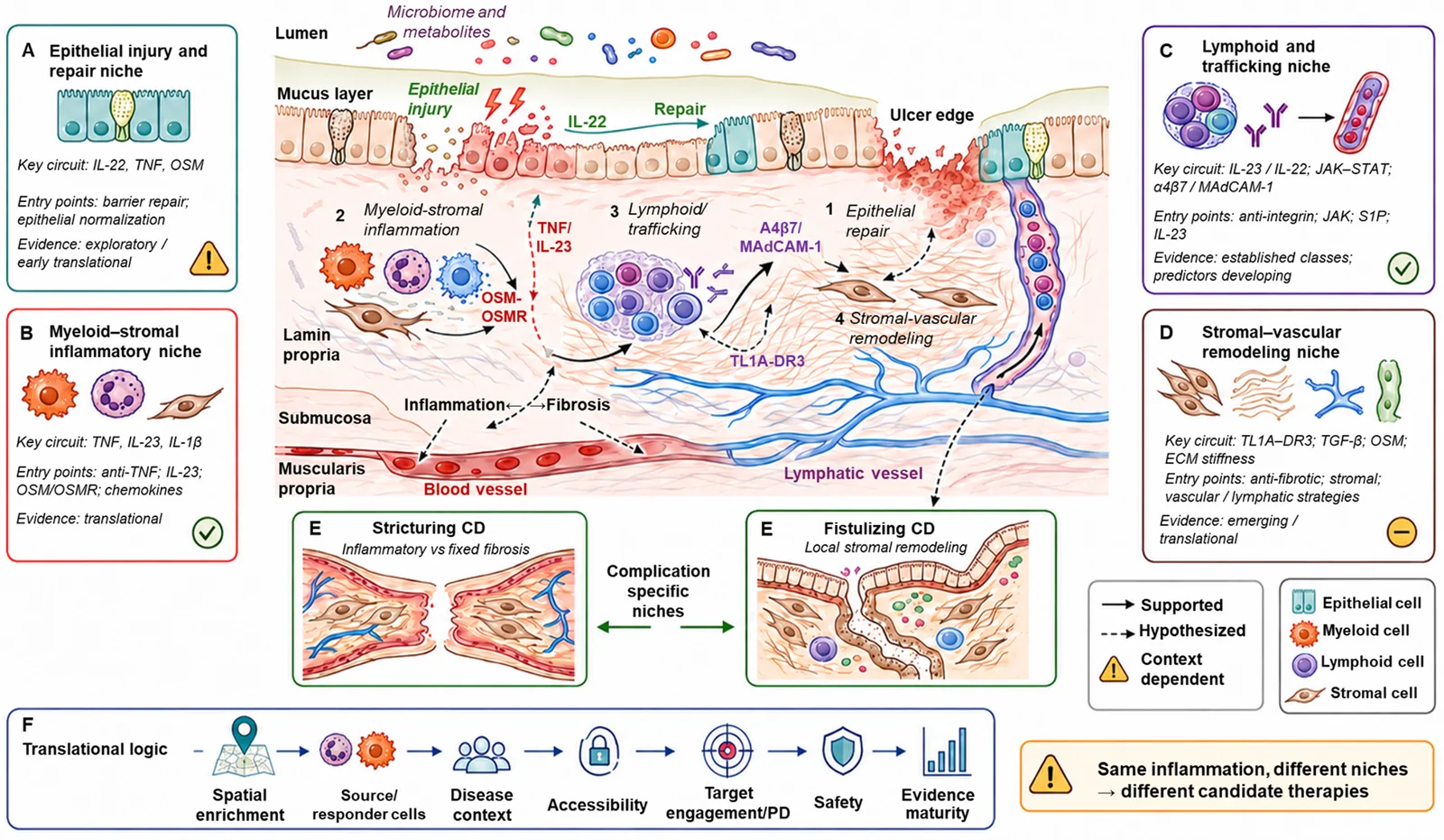
Disease-associated mucosal niches and therapeutic entry points in inflammatory bowel disease. **(A–D)** Epithelial injury and repair, myeloid–stromal inflammation, lymphoid trafficking, and stromal–vascular remodeling niches are associated with candidate signaling circuits that include IL-22, TNF, IL-23, OSM–OSMR, α4β7–MAdCAM-1, TL1A–DR3, and TGF-β and may nominate different therapeutic strategies. **(E)** Stricturing and fistulizing Crohn’s disease contain complication-specific niches that may require deeper-tissue assessment or locally directed interventions. **(F)** The working framework evaluates candidate spatially actionable targets according to seven domains: spatial enrichment, source–responder cell definition, disease context, therapeutic accessibility, measurable target engagement/pharmacodynamics, safety/selectivity, and evidence maturity. Solid arrows indicate relationships supported by human tissue or functional evidence; dashed arrows indicate inferred, proposed, or context-dependent relationships.

These considerations are applied qualitatively to both established and emerging therapeutic pathways, while the technical platforms used to identify, localize, and quantify relevant targets are compared in **Table 2**. This framework raises the threshold above bulk differential expression because a transcript elevated in averaged tissue may be diffusely expressed, may originate from a protective population, or may lie in a compartment that the proposed therapeutic modality cannot adequately reach. Spatial position can therefore alter target priority. A candidate enriched in ulcer-edge fibroblasts, fistula-associated stromal cells, epithelial repair zones, lymphoid aggregates, vascular niches, or fibrotic compartments may warrant functional testing more strongly than one identified only through average abundance, because its tissue context helps define the producing cells, responding cells, anatomical accessibility, and pharmacodynamic readouts that a therapeutic program would need to address. Representative therapeutic pathways are compared using this framework in **Table 3**, with therapeutic status distinguished from the maturity of the corresponding tissue or spatial biomarker evidence.

**Table 3:** Translational assessment of representative spatially actionable therapeutic pathways in IBD.

Target/pathway	Dominant niche or cell populations	Evidence modality	Therapeutic status	Candidate PD readout	Biomarker maturity^a^	Main limitation
TNF	Myeloid–stromal inflammatory niches; membrane-TNF+ immune cells	Tissue, single-cell, molecular imaging, and spatial proteomic evidence	Approved in UC and CD	Contraction of TNF-associated inflammatory niches; reduction in membrane-TNF+ cells	Translational	No standardized, prospectively validated spatial assay for treatment selection
IL-23	IL-23-producing myeloid cells interacting with Th17/Th1-like and ILC3 populations	Mainly tissue and single-cell evidence; limited direct spatial-response evidence	Approved IL-23-directed therapies in IBD	Reduction in IL-23-associated myeloid and lymphoid programs	Early discovery	Predictive evidence remains mainly tissue based and lacks standardized spatial validation
α4β7–MAdCAM-1	Vascular–lymphocyte interface and gut-associated lymphoid tissue	Tissue architecture, single-cell, and spatial multi-omics evidence	Anti-α4β7 approved in UC and CD; direct MAdCAM-1 blockade investigational	Remodeling of trafficking-associated lymphoid and vascular niches	Translational	Limited response cohorts and no standardized predictive spatial assay
JAK–STAT	Distributed epithelial, myeloid, lymphoid, and stromal inflammatory niches	Strong pathway-level and single-cell evidence; limited treatment-specific spatial evidence	Approved JAK inhibitors in IBD	Reduction in compartment-specific STAT-associated inflammatory programs	Hypothesis-generating	Broad pathway effects make treatment-specific spatial readouts difficult to define
S1P signaling	Lymphoid and trafficking-associated compartments	Strong clinical evidence; limited direct mucosal spatial-response evidence	Approved for UC	Redistribution of mucosal lymphocyte populations and trafficking-associated states	Hypothesis-generating	Mucosal changes may reflect downstream trafficking effects rather than direct target engagement
TL1A–DR3	Immune–stromal and fibroblast-remodeling niches	Human clinical and tissue mechanistic evidence; limited predictive spatial validation	Phase III development in UC and CD	Reduction in TL1A-responsive inflammatory–stromal and remodeling programs	Hypothesis-generating	Therapeutic development is more advanced than spatial biomarker validation
OSM–OSMR	OSM-producing myeloid cells and OSMR+ stromal/endothelial populations	Mainly tissue transcriptomic, cellular, and histological evidence with spatial contextualization	Clinically unvalidated target; prior CD Phase II program withdrawn	Reduction in OSM-associated myeloid–stromal niche activity	Translational	Strong response association but no validated OSM-directed therapy or locked spatial assay

^a^Biomarker maturity refers to the maturity of the tissue/spatial biomarker, not the therapeutic target. Categories follow the working framework in Section 6.2: translational, supported by replicated human evidence but lacking prospective assay-locked clinical validation; early discovery, supported by limited human cohorts requiring independent replication and technical validation; and hypothesis-generating, biologically plausible but lacking sufficient treatment-specific evidence. No spatial biomarker is currently established for routine IBD treatment selection. Evidence modality distinguishes tissue/bulk profiling, dissociated single-cell analysis, and directly spatially resolved measurements. Candidate PD readouts are proposed translational applications rather than validated clinical endpoints.

### 5.2 Established targets viewed through a spatial lens

Spatial context does not change whether TNF, IL-23, JAK–STAT, trafficking pathways, TL1A, or OSM are biologically relevant in IBD; it changes how these pathways are prioritized, localized, and read pharmacodynamically. For established targets, the practical question is which tissue niche carries the pathway and whether that niche predicts response.

TNF remains one of the most extensively validated targets in IBD, yet its tissue distribution is heterogeneous. Membrane-bound TNF expression within mucosal immune cells has been associated with anti-TNF responsiveness, suggesting that differences in local cellular organization may contribute to treatment heterogeneity (119). Spatial studies indicate that TNF activity is often embedded within pathogenic myeloid–stromal circuits and may intersect with OSM-associated inflammatory programs, raising the possibility that the location and cellular source of TNF signaling are clinically relevant determinants of response (31, 103).

A similar concept applies to the IL-23 pathway. Both IL-12/23p40 and selective IL-23p19 inhibitors target myeloid-derived IL-23 signaling that supports pathogenic Th17, Th1-like Th17, and ILC3 programs, and their efficacy is supported by robust clinical evidence (12–15, 120). However, accumulating data suggest that the dominant IL-23-producing niche may vary between patients, while elevated mucosal IL23A expression has been linked to improved treatment responsiveness (121, 122). These observations support the notion that spatial context may help explain why biologically similar patients exhibit different therapeutic outcomes.

Other approved and investigational therapeutic approaches can likewise be interpreted through a spatial framework. JAK inhibitors suppress JAK-dependent signaling downstream of multiple cytokine receptors and may therefore affect several inflammatory tissue niches (16, 17). S1P receptor modulators, which are approved for ulcerative colitis, reduce lymphocyte egress from lymphoid tissues, whereas anti-α4β7 integrin therapy, approved for both ulcerative colitis and Crohn’s disease, blocks gut-selective lymphocyte trafficking at the α4β7–MAdCAM-1 axis (18, 19, 110, 123). Direct MAdCAM-1 blockade remains investigational and acts at the vascular endothelial side of this trafficking pathway (124, 125). The TL1A/DR3 pathway is particularly notable because it links immune activation with stromal and fibroblast responses implicated in tissue remodeling, making it potentially relevant to both inflammatory and fibrostenotic disease states, although much of the mechanistic evidence for fibrosis remains preclinical or early translational (20, 126). Anti-TL1A therapeutic development has since advanced to Phase III in both UC and CD (NCT06052059; NCT06430801), whereas spatially resolved biomarkers for treatment selection remain at an early stage of validation. Similarly, the OSM/OSMR axis represents a biologically compelling myeloid–stromal pathway and a candidate biomarker of anti-TNF non-response. However, OSM-directed therapy has not been clinically validated in IBD; a planned Phase IIa/IIb trial of an anti-OSM monoclonal antibody in Crohn’s disease was withdrawn before enrollment (NCT04151225).

IL-17 signaling provides an important reminder that pathway activation does not necessarily imply therapeutic vulnerability. Despite strong biological rationale, IL-17 blockade failed to demonstrate benefit in Crohn’s disease and, in some settings, exacerbated intestinal inflammation (127). This experience highlights the need to consider the context-dependent and potentially protective functions of immune pathways within specific tissue compartments. Although spatial analysis cannot eliminate such risks, they may improve mechanistic understanding by identifying the cellular populations that depend on a given pathway and by revealing potential consequences of pathway inhibition before large-scale clinical testing. The relative therapeutic and biomarker maturity of these pathways is summarized in **Table 3**.

### 5.3 Emerging and exploratory niche-derived target classes

Reading targets from niches rather than from gene lists broadens the repertoire, and these classes are best kept separate from established targets because their evidence is earlier and their modalities less proven.

Epithelial repair and barrier niches point to barrier-restoring and repair-promoting targets, an area where computational and AI-guided approaches have already nominated barrier-protective candidates (100, 101). IL-1-driven stromal-neutrophil circuits and macrophage-fibroblast neighborhoods point to IL-1 family and chemokine inhibition and to the OSM axis as stromal-immune targets (102, 103). Matrix-remodeling compartments point to matrix modifiers and anti-fibrotic approaches, where the central unsolved problem is distinguishing reversible from fixed fibrosis before treatment (44, 46, 114). Fistula and stricture tissue point to stromal reprogramming and local tissue remodeling (44, 45). Microbiota-adjacent niches point to metabolite pathways, including bile acid, short-chain fatty acid, and tryptophan-aryl hydrocarbon receptor signaling, and to barrier support (82, 83). Candidate modalities across these classes span cytokine and chemokine inhibitors, matrix-modifying agents, epithelial repair enhancers, anti-fibrotics, immune-reprogramming strategies, microbial-metabolite-directed interventions, and locally delivered therapeutics designed to reach a particular compartment.

These remain target hypotheses at varying maturity. The stromal and fibrotic classes are well supported biologically by spatial atlases but clinically unproven, and claims about anti-fibrotic or fistula-closing drugs should stay cautious until reversibility and the right patient population are defined.

### 5.4 Translational models for spatially informed drug development

Spatial findings become drug-development assets only when they can be perturbed, and no single model reproduces an intact niche, so the realistic strategy is to match the model to the question. Patient-derived organoids capture epithelial behavior and can test barrier- and repair-targeted interventions, while organoid cultures and biobanks have revealed disease-imprinted epithelial states, somatic epithelial adaptation under chronic inflammation, and molecular subtypes relevant to personalized therapy (128–131). Co-culture systems add immune and stromal partners, which is necessary because the most therapeutically interesting niches are defined by interaction rather than by any single lineage. Organoid systems incorporating an autologous tissue-resident immune compartment move closer to modeling these circuits *in vitro* (132), and pediatric organoid studies have linked epithelial metabolic dysfunction to disease (133). Gut-on-chip platforms introduce flow, barrier mechanics, and microbial exposure, and inflammation-on-chip and IBD-on-chip systems have been built to model mucosal inflammation and therapeutic response (134, 135). Ex vivo mucosal explants and biopsy challenge assays retain native tissue complexity for short-term pharmacodynamic testing (136), and precision-cut intestinal slices preserve architecture across compartments for short-term perturbation (137).

The practical pairing is to use organoids for epithelial repair, co-cultures and explants for immune-stromal circuits, and chips and stromal-rich systems for fibrosis and fistula biology. Used this way, models convert a spatial correlation into a testable and, ideally, reversible mechanism, which is the step that separates a niche-derived hypothesis from a credible target.

### 5.5 Spatial pharmacodynamics and mechanism-of-action validation

Spatial assays can report on a drug’s action rather than only on a target’s existence. The relevant question shifts from whether inflammation decreased to whether the pathogenic niche was remodeled. This may include contraction of inflammatory fibroblast neighborhoods, recovery of epithelial–stromal repair crosstalk, collapse of pathogenic myeloid clusters, changes in lymphoid architecture, and resolution of cytokine or metabolite gradients. Longitudinal single-cell and spatial profiling around treatment has begun to make these readouts concrete, distinguishing baseline predictors from treatment-induced change and showing which cellular compartments move when a drug works (138–140).

This is also where spatial pharmacology becomes practical. A drug helps only if its target sits in a compartment the drug reaches, so receptor localization, antibody penetration, small-molecule distribution, and local target engagement can differ across ulcerated, fibrotic, and healed regions. Mass spectrometry imaging can in principle co-localize drug, target, and downstream pathway suppression in the same tissue (80). Treating niche normalization as a pharmacodynamic endpoint offers an earlier and more mechanistic signal than late clinical outcomes, provided the assays can be standardized.

### 5.6 From spatial association to a validated target

Spatial context clearly refines the biology of established pathways, and a handful of niche-derived circuits, OSM and the IL-1–stromal–neutrophil axis chief among them, are reproducibly tied to treatment outcome (102–104). What this does not yet establish is whether engaging such a target reverses disease; association in tissue is not evidence that inhibition helps, and the IL-17 experience stands as a warning against assuming that it does (127). The discipline this imposes on target discovery is to route candidates through the actionable-target framework and through perturbation in fit-for-purpose models before committing to a drug program, rather than moving directly from an atlas to a clinical hypothesis. The decisive evidence is functional, ideally loss- and gain-of-function data in human-relevant systems, coupled with early-phase trials that embed spatial pharmacodynamic readouts so that mechanism can be confirmed in tissue instead of inferred from clinical response.

## 6 Spatial biomarkers for therapeutic stratification, response prediction, and trial design

### 6.1 Why biomarker-guided therapy remains limited in IBD

Despite a strong rationale, biomarker-guided treatment selection is not yet routine in IBD, and the reason is instructive. Most established and candidate markers predict inflammatory burden better than mechanism-specific drug response, so they tell a clinician how active disease is without telling them which drug will work, a gap repeatedly emphasized in work on predicting disease course and treatment response (28, 141, 142). Population-level trials demonstrate average efficacy, but average efficacy does not transfer cleanly to an individual whose dominant niche may differ from the trial mean. Early mucosal gene signatures associated with infliximab response in ulcerative colitis showed that tissue can carry predictive information, and cell-centered meta-analysis later identified baseline cellular predictors of anti-TNF non-response across cohorts (140, 143). The gap since then has been less about the existence of signal than about turning it into reproducible, assay-ready, prospectively validated tools.

### 6.2 A working classification of spatial biomarker maturity

Because enthusiasm for tissue biomarkers can exceed the strength of the available evidence, we use a pragmatic four-category framework to calibrate biomarker claims. Clinically established biomarkers are analytically and clinically validated and routinely used to guide treatment selection; no spatial biomarker currently meets this standard in IBD. Translational but not routine biomarkers are supported by multiple human studies and a plausible biological mechanism but lack prospective validation using a locked assay or evidence of clinical utility. Early discovery candidates arise from one or a small number of cohorts and require independent replication, demonstration of technical transferability, and evaluation using prespecified thresholds. Hypothesis-generating candidates are biologically plausible signals derived from exploratory tissue studies, preclinical models, or cross-disease comparisons but have not been adequately assessed as treatment-response biomarkers in IBD.

Assignment within this framework is based pragmatically on several dimensions: replication across independent cohorts, external validation, availability of longitudinal treatment-response data, assay standardization and transferability, use of prespecified thresholds, and prospective evidence of clinical utility. Evidence modality is considered separately, because support from bulk tissue or dissociated single-cell analyses does not by itself establish a spatial biomarker. These categories are intended to calibrate translational maturity rather than serve as a formal regulatory classification, providing a practical basis for distinguishing more advanced translational evidence from preliminary observations and keeping biomarker claims proportionate to the supporting data. Accordingly, a candidate signal may justify evaluation as an exploratory enrichment marker or pharmacodynamic readout without yet providing sufficient evidence for routine treatment selection.

### 6.3 Spatial signatures of response and non-response

Anti-TNF therapy provides the most mature example of tissue-based response biology, although the underlying evidence spans different modalities. Single-cell analysis identified a pathogenic cellular module enriched in inflammatory mononuclear phagocytes, fibroblasts, activated T cells, and plasma cells that was associated with anti-TNF resistance in Crohn’s disease and subsequently detected across independent cohorts (31). Separately, the original OSM study evaluated mucosal OSM expression across multiple human IBD datasets, including anti-TNF-treated cohorts, supporting a reproducible association with treatment non-response while remaining primarily tissue-transcriptomic rather than a prospectively validated spatial assay (103). A biologically coherent myeloid–stromal OSM–OSMR circuit provides additional mechanistic support (104). Complementary tissue gene-expression studies have also identified transcriptomic signatures associated with anti-TNF response (144), while longitudinal single-cell profiling has distinguished pretreatment features from treatment-associated cellular changes (138). Together, these findings provide strong tissue-level and single-cell evidence for response-associated biology, but none currently constitutes a prospectively validated spatial treatment-selection assay. Within the proposed maturity framework, these signals are therefore best regarded as translational but not routinely used biomarker candidates.

For other therapeutic classes, the evidence is less mature and should likewise be separated by modality. Mucosal IL23A expression has been associated with response to IL-12/23 blockade, and biopsy studies have demonstrated treatment-induced molecular changes during selective IL-23 inhibition (121, 122). These findings are primarily tissue-based rather than prospectively validated spatial-response signatures; within the present framework, IL-23-related biomarkers are therefore classified as early discovery candidates. In contrast, anti-integrin therapy has been examined using single-cell and spatially resolved tissue approaches, providing more direct evidence of treatment-associated tissue remodeling, although validation cohorts remain limited (110, 139). JAK–STAT- and S1P-related tissue signatures remain largely hypothesis-generating for treatment-specific spatial prediction. At present, spatial biomarkers have not been prospectively validated for routine treatment selection in IBD.

### 6.4 From mucosal healing to molecular and spatial niche normalization

Treat-to-target endpoints have deepened over time, from clinical remission to endoscopic healing, histologic healing, quantified through validated histologic indices, and, in Crohn’s disease, transmural healing, with growing interest in molecular healing, supported by evidence that tighter, target-driven control improves outcomes (8, 145–148). Spatial data suggest a further layer worth defining as an exploratory endpoint: niche normalization, meaning restoration of epithelial repair, stromal quiescence, normalized immune neighborhoods, reduced pathogenic myeloid-stromal interaction, and resolved cytokine gradients. The clinical rationale is that residual fibroblast, plasma cell, macrophage, or epithelial-stress niches may persist after endoscopic healing and may explain relapse despite apparent response, in line with conserved molecular trajectories of colitis activity and resolution (111, 118, 149). Niche normalization should complement, not replace, validated endpoints, and its prognostic value has to be demonstrated longitudinally before it can be used. At present, niche normalization should be regarded as a hypothesis-generating exploratory endpoint. Its value as a pharmacodynamic or prognostic measure will require longitudinal studies demonstrating that changes in defined spatial features are reproducible, treatment-responsive, and independently associated with subsequent clinical outcomes.

These tissue-level readouts are also complementary to imaging rather than competing with it: mucosal spatial omics is better suited to resolving local cellular mechanisms, whereas cross-sectional imaging and radiomics better capture transmural, mesenteric, and structural disease burden. A similar translational strategy may apply to less invasive blood- or stool-derived measurements, including circulating proteins, RNA, metabolite profiles, or composite biomarker panels. Rather than replacing tissue profiling, such assays could serve as scalable surrogates for spatially defined tissue states if they are validated against paired tissue measurements and longitudinal clinical outcomes. A workable precision-IBD framework will therefore align mucosal spatial readouts with imaging, blood, stool, clinical, and longitudinal outcome data rather than rely on any single layer.

### 6.5 Spatially informed treatment sequencing and combination therapy

If patients could be characterized by dominant niche rather than by diagnosis alone, sequencing decisions might become more rational. A myeloid-stromal inflammatory pattern might favor mechanisms that engage that circuit, whereas a trafficking-associated pattern might favor anti-integrin or S1P approaches. Epithelial-repair-defective states may require barrier support alongside immune suppression, while fibrotic or fistulizing patterns may require stromal-directed or local strategies that current anti-inflammatory drugs do not provide. Mixed inflammatory-fibrotic states, which are common, might justify rational combinations. These remain conceptual proposals meant to structure hypotheses, and each would need prospective evaluation before it could influence care. Their purpose is to generate testable hypotheses linking tissue states to mechanism-specific benefit, not to rename existing clinical phenotypes.

### 6.6 The immediate role: enrichment, pharmacodynamics, and adaptive trials

The most credible immediate role for spatial biomarkers is inside clinical trials, especially as enrichment and pharmacodynamic tools (**Figure 4**), and the realistic uses are worth specifying. Spatial signatures can enrich trial populations for the mechanism a drug targets, increasing the chance of detecting a true effect. They can also define early pharmacodynamic endpoints, reclassify responders and non-responders for subgroup analysis, support exploratory companion-diagnostic development, and underpin adaptive designs that drop futile arms early or adjust therapy by tissue response. The TL1A programs are a useful template, since modern TL1A development has paired the drug with biomarker-based patient selection from early phases (20, 126), and OSM, IL-23, integrin, anti-TNF, fibrotic Crohn’s disease, and fistula-related programs offer analogous opportunities. Importantly, biomarker maturity should determine its intended role: hypothesis-generating and early discovery signals are most appropriate for exploratory analysis, whereas translational candidates may support prospective enrichment or pharmacodynamic testing without yet being used for routine treatment allocation.

**Figure 4:**
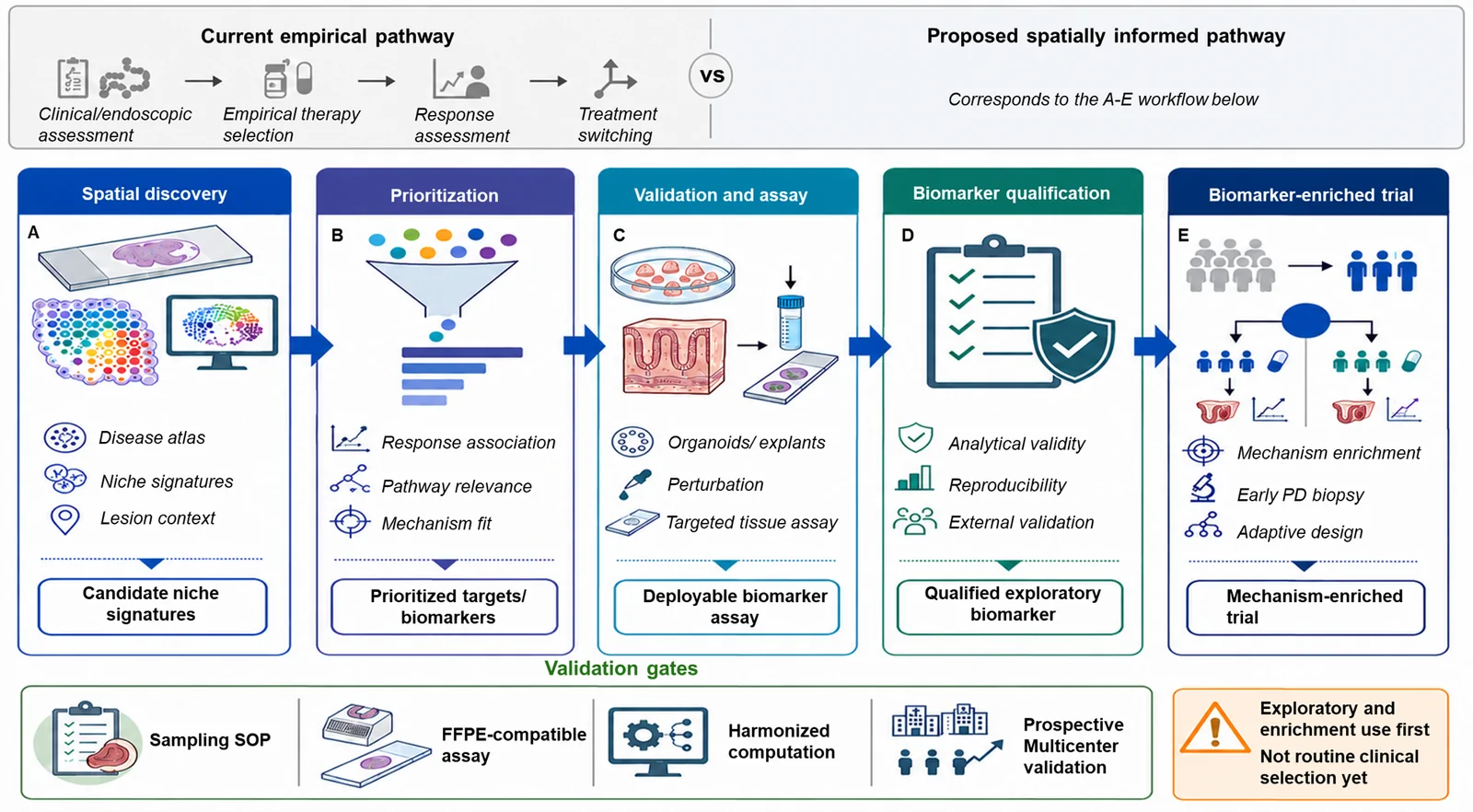
From the current empirical treatment pathway to a proposed spatially informed roadmap for biomarker-enriched trials in inflammatory bowel disease. The current pathway relies largely on clinical/endoscopic assessment, empirical therapy selection, response assessment, and treatment switching, whereas the proposed spatially informed pathway follows the **(A**–**E)** workflow. **(A)** Spatial atlases define candidate niche signatures and lesion context. **(B)** Targets and biomarkers are prioritized by response association, pathway relevance, and mechanistic fit. **(C)** Functional testing supports translation into deployable tissue assays. **(D)** Candidate assays undergo analytical validation, reproducibility testing, and external validation. **(E)** Validated exploratory biomarkers may support mechanism-based enrichment, early pharmacodynamic assessment, and adaptive trial design. Progression requires standardized sampling, FFPE-compatible assays, harmonized computation, and prospective multicenter validation. Spatial biomarkers remain primarily exploratory, enrichment, and pharmacodynamic tools rather than routine treatment-selection assays.

As an illustrative proof-of-mechanism design, a prospective anti-TL1A study could incorporate baseline biopsies to characterize an exploratory OSM-associated myeloid–stromal niche, followed by a predefined on-treatment biopsy to quantify niche contraction or remodeling as a pharmacodynamic endpoint. Clinical and endoscopic outcomes, CRP, and fecal calprotectin would be assessed in parallel to determine whether the tissue readout provides incremental mechanistic or predictive information. This example is hypothesis-generating only: OSM-associated niche status has not been validated as a predictive biomarker for TL1A blockade and should not currently guide treatment allocation.

Maturing any of these into a companion diagnostic requires meeting the usual standards of analytical validity, clinical validity, and clinical utility, with assays that are locked, interpretable, and prospectively tested (150). Claims of companion-diagnostic readiness would be premature, and practical factors, including FFPE compatibility, turnaround time, and cost, will determine which signatures can be deployed at scale.

### 6.7 What mucosal tissue can and cannot yet predict

Mucosal tissue demonstrably carries information about anti-TNF response, with the pathogenic myeloid–stromal module and OSM the strongest and most often replicated examples (31, 103, 104, 143). Whether any spatial signature predicts response prospectively, and at a threshold sharp enough to guide an individual decision, remains unresolved, because almost all current evidence is retrospective and cross-sectional. Current evidence therefore supports spatial biomarkers primarily as discovery, enrichment, and pharmacodynamic tools rather than as routine treatment-selection assays. Spatial markers should accordingly be evaluated as complementary biomarkers that resolve mechanism and tissue compartment rather than as replacements for established measures such as CRP or fecal calprotectin. Their clinical value will depend on demonstrating reproducible incremental value beyond established clinical, serum, stool, endoscopic, histological, and imaging variables. Progression from an exploratory tissue signature to a clinically actionable biomarker will require an assay-locked platform, prespecified thresholds, and prospective external validation.

## 7 Discussion and roadmap: from spatial mucosal immunology to precision IBD trials

Recent advances in single-cell and spatial technologies suggest that the biologically relevant unit of analysis in inflammatory bowel disease may extend beyond the inflamed biopsy to the mucosal immune niche. By defining how epithelial, immune, stromal, vascular, neural, and microbiome-associated compartments are spatially arranged and functionally connected, mucosal profiling provides a framework for understanding disease heterogeneity as a set of organized tissue states rather than as uniform inflammation.

Accumulating evidence indicates that distinct cellular neighborhoods are associated with inflammatory, fibrotic, reparative, and treatment-responsive states. However, translating these observations into precision IBD trials remains constrained by pre-analytical variability, cohort heterogeneity, computational reproducibility, assay scalability, and regulatory requirements for biomarker qualification. The central challenge is therefore not only to generate increasingly detailed spatial datasets, but also to convert spatial discoveries into analytically validated, clinically testable biomarkers. The following sections outline a staged roadmap from tissue standardization and platform readiness to external validation, biomarker qualification, and spatially informed precision trials.

### 7.1 Pre-analytical standardization: sampling, pairing, annotation, and metadata

The reliability of spatial profiling depends heavily on pre-analytical tissue quality, and IBD sampling is particularly vulnerable to variation. Standardized biopsy protocols should define the number, size, orientation, and handling of samples needed to preserve the crypt–surface axis, while recognizing that biopsy depth limits access to the submucosal and transmural processes that are central to Crohn’s disease complications. Limited biopsy size further restricts the number of spatial niches that can be sampled within a heterogeneous lesion, creating a risk that a small mucosal fragment may not represent the dominant disease process. This limitation is particularly important in fibrostenotic and fistulizing Crohn’s disease, where relevant pathology extends beyond the mucosa into submucosal and transmural compartments. Where feasible, paired inflamed and non-inflamed sampling should be incorporated because predictive or residual-disease signals may also be detectable in tissue that appears endoscopically normal, whereas sampling only the most severely inflamed area may provide an incomplete view of disease heterogeneity. Anatomical site annotation should record segment and sampling position precisely, as the ileum, colon, and rectum represent distinct tissue contexts and spatial niches are anatomically dependent. Treatment-exposure metadata should likewise capture prior and current drug class, dose, timing, and response, because prior therapy can reshape the niches being measured, and unannotated treatment heterogeneity may create, obscure, or distort spatial signals. These pre-analytical requirements may appear procedural, but they are fundamental to the reproducibility and external validation of spatial signatures across studies (43, 69).

### 7.2 Assay and platform readiness: FFPE compatibility and benchmarking

Two technical considerations will strongly influence whether discovery-scale spatial profiling can be extended to clinical cohorts. FFPE-compatible assays matter because archival blocks and trial specimens are overwhelmingly formalin-fixed, so a method that requires fresh or frozen tissue cannot use the cohorts that would validate it; FFPE-capable imaging-based and probe-based platforms are therefore strategically important even where fresh-tissue methods offer more breadth (68, 72, 73). Cross-platform benchmarking matters because the same tissue can yield different cell calls on different platforms, and harmonized metrics, shared reference data structures, and inter-platform comparisons are needed before a signature defined on one system can be trusted on another (96, 97, 99). Until benchmarking matures, a signature should be reported with the platform on which it was derived and, ideally, confirmed on a second.

### 7.3 Clinical heterogeneity, cohorts, and external validation

The same biological heterogeneity that makes spatial profiling informative also complicates cohort design. Disease location and behavior, pediatric versus adult onset, treatment-naive versus treatment-exposed status, prior surgery, complications, and disease duration all reshape tissue, so a signature discovered in one context may not transfer to another, and real-world diversity in ancestry, diet, and microbiome adds further variation (43, 46, 47). Cohorts therefore need to be designed around these axes from the outset, with enough representation in each stratum to support context-specific claims. External validation cohorts are essential, as models evaluated only through internal cross-validation on small datasets are prone to overfitting, and the credibility of the field depends on demonstrating that spatial signatures remain robust in independent populations, ideally assembled and analyzed by different research groups (73, 97).

### 7.4 Computation, reproducibility, and explainable models

Computation is where many spatial findings will either become robust or fail to replicate. Harmonized pipelines, shared reference atlases, transparent feature selection, and reproducible reporting are needed to make results comparable, and standardized data structures and benchmarking efforts are early steps (96, 97). Models that link spatial features to outcomes must be interpretable, because a clinician or regulator cannot act on a prediction whose basis is opaque, and the same interpretability requirement applies to digital-pathology models intended as scalable surrogates for spatial signatures (84–86). Data sharing under FAIR principles, supported by harmonized clinical, histological, endoscopic, and pre-analytical metadata, would accelerate this work (151).

### 7.5 Regulatory translation: from signature to qualified biomarker

Moving from discovery to clinical use requires meeting evidentiary standards that spatial biomarkers have not yet met. Biomarker qualification frameworks distinguish analytical validity, clinical validity, and clinical utility. A spatial biomarker must therefore show that the assay is reliable, that the measure relates to a relevant clinical outcome, and that its use improves decision-making or outcomes before it can guide therapy (150, 152). The most plausible regulatory path is sequential: spatial signatures may first enter trials as exploratory or enrichment tools, then be translated into feasible derivative assays, and only later, if prospectively validated, inform routine decisions. Trial enrichment and adaptive designs are suitable frameworks because they can use candidate biomarkers to improve trial efficiency while generating the prospective evidence required for qualification (153). Full spatial multi-omics is unlikely to become the endpoint of routine clinical testing; the more realistic goal is to qualify scalable derivative assays that retain the clinically relevant information of spatial signatures.

### 7.6 A staged path toward spatially informed IBD trials

A realistic translational path should move spatial mucosal immunology from discovery-scale profiling to clinically testable biomarkers through staged validation. The first step is to establish disease- and context-specific reference atlases that capture major IBD phenotypes, including ileal and colonic Crohn’s disease, ulcerative colitis, fibrostenotic and fistulizing complications, pediatric disease, and postoperative recurrence. These atlases should be built on standardized tissue acquisition, anatomical annotation, treatment-exposure metadata, and independent cohort replication (43, 154).

The next step is to convert high-dimensional spatial signatures into analytically tractable assays that can be applied to routinely available specimens, particularly formalin-fixed paraffin-embedded tissue. Targeted RNA, protein, multiplex imaging, or digital pathology panels may preserve the most informative spatial features while enabling larger retrospective and prospective studies (84, 86). In a large multicenter trial, comprehensive high-plex spatial profiling would more realistically be applied to a discovery or mechanistic subset rather than to every participant. The resulting signature could then be translated into a reduced, locked targeted RNA, protein, multiplex-imaging, or digital-pathology assay for deployment across the broader trial population after analytical validation. The optimal platform for spatial discovery is therefore not necessarily the optimal platform for clinical deployment. Analytical validity, concordance with the original spatial signature, and external validation should be established before prospective clinical testing. Such assays should then be linked to biologically informed pathotypes, pharmacodynamic endpoints, and response-associated niche remodeling, rather than simply reproducing existing clinical classifications.

The final stage is prospective testing in biomarker-enriched or adaptive IBD trials. In this setting, spatially derived biomarkers could support patient enrichment, mechanism-based treatment allocation, early futility assessment, therapeutic sequencing, and rational combination strategies (153). However, routine clinical use will require evidence that a spatial-biomarker-guided decision improves outcomes beyond existing clinical, endoscopic, histologic, and molecular markers, with sufficient added value to justify its complexity and cost. Until such evidence is available, spatial multi-omics should be viewed as a powerful engine for mechanistic discovery, pharmacodynamic assessment, and precision trial design, rather than as a routine clinical test.

### 7.7 Broader relevance to other mucosal inflammatory diseases

The intestinal niche framework also gains perspective from chronic inflammatory diseases at other mucosal sites. In chronic rhinosinusitis with nasal polyps, the upper-airway mucosa exhibits epithelial barrier dysfunction, type 2 inflammation, eosinophil and mast-cell activation, fibroblast remodeling, and marked endotype heterogeneity (155, 156). Human epithelial progenitor studies further suggest that allergic inflammation can leave a durable epithelial memory that may sustain barrier dysfunction (157), while single-cell and spatial transcriptomic analyses have identified disease-specific epithelial, immune, and stromal states, including immune–epithelial interactions, progenitor and tuft-cell programs, macrophage–eosinophil recruitment, and remodeling associated with polyp formation (158, 159). These findings support a broader mucosal principle relevant to IBD: chronic inflammation reflects organized cellular neighborhoods, epithelial injury memory, incomplete repair, and stromal remodeling rather than bulk cytokine abundance alone. However, the intestine and upper airway differ substantially in anatomy, microbial exposure, dominant immune programs, and therapeutic endpoints. These cross-organ comparisons are intended as conceptual and methodological analogies rather than evidence of shared therapeutic mechanisms or interchangeable biomarkers.

## 8 Conclusion

Crohn’s disease and ulcerative colitis are spatially organized as well as molecularly heterogeneous, and mucosal immune niches provide a tissue-level framework for linking mechanisms to therapeutic targets, pharmacodynamic readouts, and patient stratification. Across epithelial injury and repair, myeloid and lymphoid inflammation, stromal and matrix remodeling, vascular and lymphatic change, and host–microbe interfaces, spatial multi-omics can identify disease-relevant circuits that are obscured in averaged tissue profiles. However, most spatial biomarkers remain at the discovery or early translational stage and should currently be viewed as tools for mechanistic hypothesis generation, pharmacodynamic assessment, and biomarker-enriched trial design rather than routine treatment selection. The immediate priority is therefore rigorous validation of reproducible, scalable, and clinically interpretable spatial signatures in longitudinal and multicenter cohorts, rather than the continued expansion of increasingly complex atlases. With systematic validation, spatial profiling may ultimately transform the inflamed biopsy from a descriptive measure of disease activity into a mechanism-oriented substrate for precision drug development in IBD.
